# Dipolar Relaxation of Water Protons in the Vicinity of a Collagen-like Peptide

**DOI:** 10.1021/acs.jpcb.2c00052

**Published:** 2022-03-26

**Authors:** Jouni Karjalainen, Henning Henschel, Mikko J. Nissi, Miika T. Nieminen, Matti Hanni

**Affiliations:** † Research Unit of Medical Imaging Physics and Technology, University of Oulu, P.O. Box 5000, Oulu 90014, Finland; ‡ Department of Applied Physics, University of Eastern Finland, Kuopio 70210, Finland; ¶ Department of Diagnostic Radiology, Oulu University Hospital, Oulu 90014, Finland; § Medical Research Center, University of Oulu and Oulu University Hospital, Oulu 90014, Finland

## Abstract

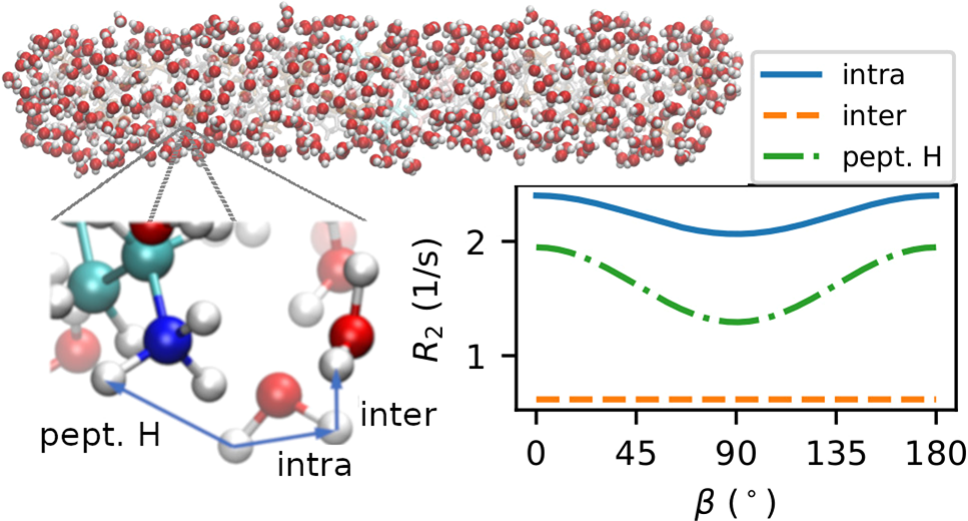

Quantitative magnetic resonance imaging is one of the few available methods for noninvasive diagnosis of degenerative changes in articular cartilage. The clinical use of the imaging data is limited by the lack of a clear association between structural changes at the molecular level and the measured magnetic relaxation times. In anisotropic, collagen-containing tissues, such as articular cartilage, the orientation dependency of nuclear magnetic relaxation can obscure the content of the images. Conversely, if the molecular origin of the phenomenon would be better understood, it would provide opportunities for diagnostics as well as treatment planning of degenerative changes in these tissues. We study the magnitude and orientation dependence of the nuclear magnetic relaxation due to dipole–dipole coupling of water protons in anisotropic, collagenous structures. The water–collagen interactions are modeled with molecular dynamics simulations of a small collagen-like peptide dissolved in water. We find that in the vicinity of the collagen-like peptide, the dipolar relaxation of water hydrogen nuclei is anisotropic, which can result in orientation-dependent relaxation times if the water remains close to the peptide. However, the orientation-dependency of the relaxation is different from the commonly observed magic-angle phenomenon in articular cartilage MRI.

## 1 Introduction

Articular cartilage (AC) is the soft tissue that covers the bones at joint surfaces. It reduces friction and provides shock absorption in the joint motion. Magnetic resonance imaging (MRI) is a noninvasive method well suited to study soft tissues and holds a promise for being an efficient diagnostic tool for early degenerative changes in articular cartilage. However, the association of the molecular-level structure to quantitative MRI parameters, such as the transverse relaxation time *T*_2_, remains an open problem.

The most abundant macromolecular component in articular cartilage is type II collagen, which amounts to 20–40% of the wet weight of cartilage. Collagen consists of tropocollagens that form prototypic fibrils with a thickness of 18 ± 5 nm, which then bundle up to larger fibers with diameters typically below 100 nm in knee articular cartilage.^1^ The tropocollagen unit consists of three polypeptide strands, which form a triple-helical structure. In addition to collagen, the other important macromolecular component is proteoglycans, which are embedded in and interact with the collagen matrix. The majority of the wet weight of articular cartilage is water, which surrounds and penetrates the fibers/fibrils.^2^^1^H MRI of articular cartilage reflects the interactions of water with the macromolecular structures it surrounds.

Articular cartilage can be divided into three zones by the orientation of collagen fibrils.^2^ In the superficial zone, fibers are parallel to the surface. Deeper, in the radial zone, the fibrils are on average aligned perpendicular to the bone-cartilage interface. Between the superficial and radial zones is the transitional zone, in which the fibril orientation is more or less random. Changes in collagen orientation, especially in the superficial zone, have been linked to early osteoarthritis.^3^

The anisotropy of relaxation times in tissues containing aligned networks of collagen has been studied experimentally in cartilage^4−7^ and tendon,^4,8^ with results mostly pointing to either weak or negligible anisotropy in the longitudinal relaxation time *T*_1_. For *T*_2_-weighted images and *T*_2_ maps, the story is different: If one rotates a small cartilage sample around an axis perpendicular to the bone-cartilage interface, the *T*_2_-weighted image of the radial zone is largely unaffected. Rotations around the other axes produce systematic variations in *T*_2_-weighted MRI images, and the corresponding relaxation time maps.^6,7,9^ This phenomenon is known as the magic angle effect^10^ and in collagenous tissues it is usually attributed to nonaveraged dipolar couplings of water bound to the collagen.^8,11,12^ The orientational symmetry of *T*_2_ in the radial zone suggests that the average macromolecular environment experienced by the hydrogen atoms of water can be assumed to be cylindrically symmetric.

Competing explanations for the relaxation anisotropy include the formation of strong water bridges^11^ and anisotropic, water-filled cavities inside the tissue.^13,14^ In both models, the anisotropy in relaxation is due to residual ^1^H–^1^1H dipole–dipole couplings, although they arise for different reasons. The role of proton exchange in relaxation is brought forward, especially in studies of the longitudinal relaxation time in the presence of a continuous-wave spin-lock (*T*_1ρ_).^15^ However, the magic-angle effect is seen also in *T*_1ρ_ of articular cartilage,^9^ and a comprehensive theoretical picture of how proton exchange would create the orientation-dependence is lacking.

Most of the earlier computational work on collagen has focused on the structure and biochemistry of collagen itself.^16^ Anisotropic rotational motion of water near collagen has been analyzed previously by numerical and analytical models,^12^ as well as molecular dynamics (MD) simulations focusing on the so-called water bridges.^17,18^ Water diffusion in a collagen network has been computationally modeled with coarse-grained Monte Carlo simulations of Brownian dynamics^19^ and Langevin dynamics.^20^ Although the effect of proteins and other solutes on water dynamics is an extensively studied subject,^21−23^ these studies do not consider macromolecules in a fixed or restricted orientation condition as they appear in anisotropic tissues, such as articular cartilage. Furthermore, little, if any attention is given to the effects which could be seen in ^1^H NMR/MRI due to the anisotropic motion of water in these systems.

In this paper we study the sources of ^1^H relaxation anisotropy with MD simulations of a small collagen-like peptide dissolved in water. With our molecular model we aim to estimate how the dipolar relaxation would appear in a uniaxially anisotropic network of collagen molecules, such as the radial zone of articular cartilage. In the present work we try to avoid assumptions about the motional characteristics or specific binding sites of the water molecules and compute relaxation rates directly from the simulated data. Our approach represents a first step toward more comprehensive and realistic models of water in a collagenous environment. The validity of the methodology for computing the dipolar relaxation from MD simulations has previously been demonstrated for pure water.^24^

In the Supporting Information (SI) we derive the necessary extensions to the well-known Redfield relaxation theory^25−27^ of dipolar relaxation in isotropic liquids to handle the residual dipole–dipole couplings (RDCs) and to study the orientation dependence of relaxation with respect to the direction of the main magnetic field. Unlike in the seminal work by Woessner,^28,29^ we start our derivation from first-principles, without the assumption of how the relaxation rates depend on the spectral densities of molecular motion. We also do not assume any overall motion that would average out the RDC. Similar developments, albeit with a slightly different formalism have been given, for example, in ref (30). With the combination of theoretical work and simulations, we clarify the role of water rotation and dipolar interaction in the ^1^H relaxation times in quantitative MRI of articular cartilage and other tissues containing aligned networks of collagen.

## 2 Theory

Here we estimate the evolution of the longitudinal and transverse magnetizations in the presence of time-dependent and residual dipole couplings. The equations are derived for a pair of identical spin-1/2 nuclei. We present here mainly the results. The complete derivation can be found in the SI. The spin Hamiltonian can be split to time-independent and time-dependent parts *H*_0_ and *H*_1_(*t*) as
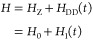
1
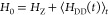
2
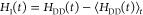
3where *H*_Z_ = ω_0_ (*I*_*z*_ + *S*_*z*_) and *H*_DD_ refer to Zeeman and dipole–dipole interactions, respectively, for the two spins *I* and *S*. The time-average ⟨*H*_DD_(*t*)⟩_*t*_ is the RDC.

The dipole–dipole coupling can be decomposed into spatial functions *F*_*m*_ and spin-operators *V*_*m*_ so that
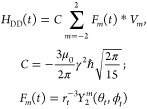
4where *r*_*t*_ = *r*(*t*), is the length of the internuclear vector and θ_*t*_ = θ(*t*) and ϕ_*t*_ = ϕ(*t*) describe the orientation of the vector with respect to the external magnetic field. In SI units *C* = −2.93083474 × 10^–24^ m^3^/s. The spin operators *V*_*m*_ with |*m*| = 0, 1, and 2 correspond to in-phase magnetization with longitudinal two-spin order, antiphase magnetizations, and double-quantum coherences, respectively:
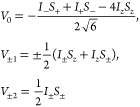
5The spatial functions *F*_*m*_ contain the normalized second rank spherical harmonics *Y*_2_^*m*^(θ, ϕ):
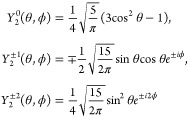
6where the spherical harmonics with different |*m*| can be interpreted as corresponding to different modes of reorientational motion. The normalization condition is
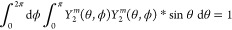
7To aid in interpretation of the molecular motion, the spherical harmonics can be written in Cartesian coordinates, here for brevity only in the hemisphere 0 < θ < π/2:
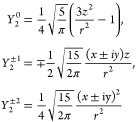
8We see that if *r* is constant as in intramolecular coupling with a rigid water molecule model, the correlation time with *m* = 0 is dependent only on the *z* coordinate, *m* = ±2 is dependent only on motion in the *x*, *y*-plane, and *m* = ±1 represents a mixed motion involving movement in-plane and also along the *z*-direction.

We assume that the motion of internuclear vectors **r** has on average uniaxial symmetry around the long axis of the collagen-like peptide. We analyze the validity of this assumption in section S7 of the SI. A coordinate system with one axis, denoted *z*′-axis, along the symmetry axis is taken as the principal axis system (PAS) of the RDC. Treating the dipolar coupling as a small time-dependent perturbation with Redfield theory (see sections S1 and S2 in the SI) gives the evolution of the longitudinal magnetization
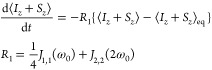
9when the correlation time(s) τ_c_ of the dipolar coupling are much shorter than the inverse of the secular RDC. The spectral densities in any coordinate system tilted with respect to the PAS by an angle β can be expressed with the spectral densities in the PAS, *J*′_*nn*_(ω), using the rotation formula (section S5 in the SI)
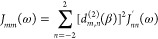
10Here, β is the angle between the symmetry axis and the main magnetic field. *d*_*m,n*_^(2)^ (β) are elements of the Wigner small-d matrix. From here on, quantities denoted with a prime (′) are computed in the coordinates in which the system is uniaxially symmetric around the *z*′-axis. The spectral densities in the PAS are
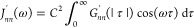
11The time-correlation functions (TCFs) *G*_*nn*_^′^(|τ|) are computed in the PAS of the RDC,
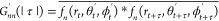
12
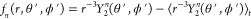
13The overline denotes ensemble average. θ′ is the angle between the axis of symmetry and the internuclear vector. ϕ′ is the other spherical coordinate in the plane perpendicular to the symmetry axis. Again, we use the assumption of a cylindrically symmetric macromolecular environment for the water molecules and choose the *x*′ and *y*′ axes in this plane arbitrarily. The subscripts *t* and *t* + τ denote the respective time points.

For transverse magnetization we find (see sections S3 and S4 in the SI) the time-evolution of the rotating frame operator *I*_*x*_*r*__ + *S*_*x*_*r*__ to be of the form
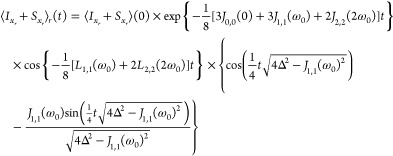
14
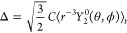
15
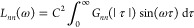
16In addition to the spectral densities encountered earlier, eq 14 contains the effect of the secular RDC in the term Δ = 3*d*_*IS*_, where *d*_*IS*_ the secular dipole–dipole coupling as defined in ref (31).

The transverse magnetization is a product of an exponential decay and two oscillating terms. In section 5.5 we see that in our case the oscillation by cos {−1/8 [*L*_1,1_(ω_0_) + 2*L*_2,2_(2ω_0_)] *t*} is much slower than the one caused by the term containing the secular RDC. It is also much slower than the relaxation and can be neglected to a good approximation.

If the RDCs are nonvanishing, the form of the evolution of the transverse magnetization depends on the sign of 4Δ^2^ – *J*_1,1_(ω_0_)^2^. If the sign is negative, the result is a multiexponential decay. If the sign is positive, the evolution is a combination of an oscillating component and an exponentially decaying factor with decay rate
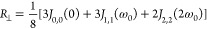
17If also the RDC vanishes (Δ = 0), the relaxation rate of the transverse magnetization becomes the familiar^25,26^
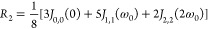
18

## 3 Modeling

### 3.1 The Molecular Model

Here we chose the trimer of the polypeptide (Pro-Hyp-Gly)_4_-Glu-Lys-Gly-(Pro-Hyp-Gly)_5_ with PDB ID: 1QSU(32) to represent a partial sequence of the triple-helical structure of a tropocollagen of type II collagen. The length of the peptide in its initial state is 8.8 nm. The typical length and diameter for a type-II collagen monomer are 1.5 and 300 nm, respectively.^1^ In articular cartilage, the diameter of a collagen fiber can be up to 200 nm. For atomistic simulation these structures are huge, which makes their simulations challenging. We can, however, quite safely assume that the most important collagen–water interactions affecting water dynamics are short-ranged. In effect, the individual water molecules mostly experience the local structure of the collagen. This should allow us to model the collagen with small representative samples of the fibrillous structure. The same may not apply for the intermolecular contribution to the residual dipolar couplings. We will discuss this limitation further in section 4.1. A weak long-ranged (∝ *r*^–3^) aligning force felt by the water molecules can be produced by the electric field of the protein.^21^ This could be reflected in the intramolecular ^1^H–^1^H dipole–dipole couplings in water. The long-range contribution from electrostatic forces is accounted by the particle mesh Ewald method as explained in section 3.2. Since the long-range forces are computed with a periodic approximation in the PME method, the size and shape of the simulation box and the proportion of water will affect the results. We did not try to separate the contributions of short- and long-range forces to the water alignment.

In simulations, the peptide was solvated in TIP4P/2005 water, which is a rigid, nonpolarizable, planar, four-site model.^33^ We assume that bond vibrations are mostly uncoupled from rotational motion and that the vibrations themselves are too fast to cause relaxation. Among the commonly used rigid, nonpolarizable water models, TIP4P/2005 has been shown to best reproduce the experimental rotational correlation times in simulations.^34^ Relatedly, it is also well suited for simulating ^1^H nuclear spin relaxation.^24^

The initial coordinates as well as the force field parameters were created with xLeap, which is part of the AmberTools15 package.^35^ The Amber force field ff14SB^35^ with rigid bonds was used to model the atomic interactions. A cutoff radius of 12 Å was used for nonbonded forces, with a switching function applied at 10 Å. Before the solvation, xLeap was used to add hydrogen atoms and missing heavy atoms to the peptide. This left the C-termini glycine residues deprotonated and the N-termini proline residues protonated.

### 3.2 Simulations

The MD simulations were performed with NAMD.^36^ Periodic boundaries were applied in all directions. For the bulk water simulation, the cubical simulation box contained 1926 water molecules and the initial box side length was 43 Å. The system with the triple-helical peptide contained 18 393 water molecules. The simulation box side lengths were initially *L*_*x*_, *L*_*y*_, *L*_*z*_ = 86.7 Å, 60.5 Å, 128.6 Å, respectively.

The simulations of both systems consisted of 1000 steps of potential energy minimization, followed by ≈18 ns of constant-temperature, constant-pressure (*NPT*) ensemble simulation at *T* = 300 K and *P* = 1 atm. The Langevin thermostat with 1/ps damping factor was used to control temperature. The Langevin-piston-inspired barostat present in NAMD^36^ was used with a 100 fs oscillation period and 50 fs decay. A time step of 1 fs was used for the integrator, and nonbonded forces were computed at each step. Long-range electrostatic forces were updated every second step with the particle mesh Ewald method. Neighbor lists with 14 Å lookup radius were used for nonbonded interactions. The lists were updated every tenth time step. The trajectory of the molecules was recorded every 100 fs. First 3 ns was discarded as equilibration, and the rest of the trajectory was used for analysis. The input files to reproduce the simulations are available for download at the Zenodo archive (10.5281/zenodo.6330600). The trajectories and log files of the simulations are available from the corresponding authors by request.

## 4 Analysis of the Time-Correlation Functions of Dipole–Dipole Couplings

We assume that on average the dipolar couplings experienced by the water molecules are cylindrically symmetric and the symmetry axis is defined by the eigenvector corresponding to the smallest eigenvalue of the inertia tensor of the peptide. The correlation functions in eq 12 were computed in the average PAS of the inertia tensor of the peptide. Then the functions were fitted with a triexponential,
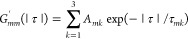
19The multiexponential correlation function leads to a spectral density
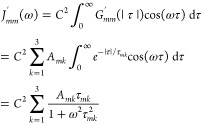
20This form can easily be used to compute the relaxation rates, once the parameters *A*_*mk*_ and τ_*mk*_ have been obtained from the fit.

Since the trajectory sampling is every 100 fs, the details of the femtosecond scale libration of the water molecules do not show up in the correlation functions. We consider the contribution of this motion to relaxation as small. If one monoexponential function in eq 20 would be dedicated to describing the libration, its amplitude *A*_*mk*_ would be small and its correlation time τ_*mk*_ very short. Other exponential terms corresponding to, for example, the overall rotation of the molecule, have both larger amplitude and longer correlation time and they dominate the spectral density of eq 20. The libration can and will affect the time-evolution of the magnetization by decreasing the RDCs.

Three exponentials were found sufficient to capture well the characteristics of the correlation functions in most cases studied here, with one exception (see section 5.7). To estimate the error made in fitting the TCFs we compared the integrals of the TCFs and their respective fits, which commonly gave a difference of 2% or less. The number of exponentials in the fit is not relevant as long as it does not substantially affect the accuracy of the fit. We do not claim that the individual τ_*mk*_ represent for example different characteristic motions. Instead, we compute the integrated correlation times
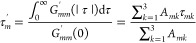
21to analyze the molecular motion in the PAS of the inertia tensor. These integrated correlation times are computed in section 5.2 and their connection to relaxation anisotropy is discussed in section 6 via the Cartesian representation of spherical harmonics [eq 8]. It would also be possible to proceed from the correlation functions to the relaxation rates by numerically computing the Fourier transformations of the correlation functions as has been done in the case of ^129^Xe relaxation due to chemical shift anisotropy.^37^ Our strategy allows the analysis of the integrated correlation times and to draw the connection from them to the relaxation rates.

**Figure 1:**
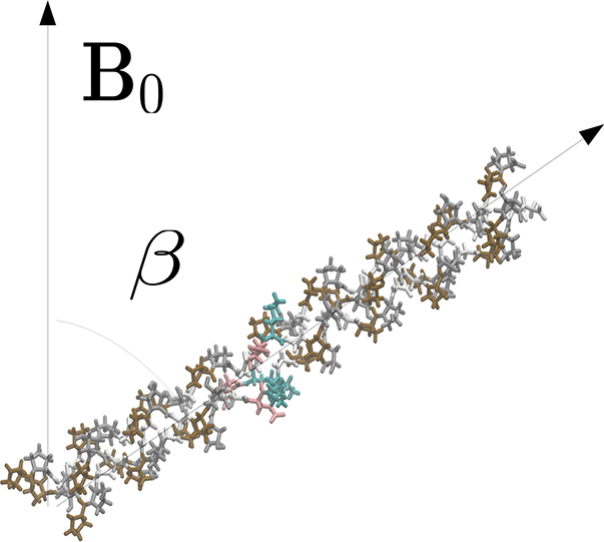
Schematic illustrating the angle β between the peptide and the main magnetic field.

The correlation functions and RDCs appearing in the relaxation eqs 9, 14, and 18 were computed in the principal axis system of the inertia of the peptide. The assumed cylindrical symmetry implies that there is only one angle needed for the transformation of results for different peptide orientations with respect to the main magnetic field. This angle β is visualized in Figure 1.

### 4.1 Water Selections

**Figure 2:**
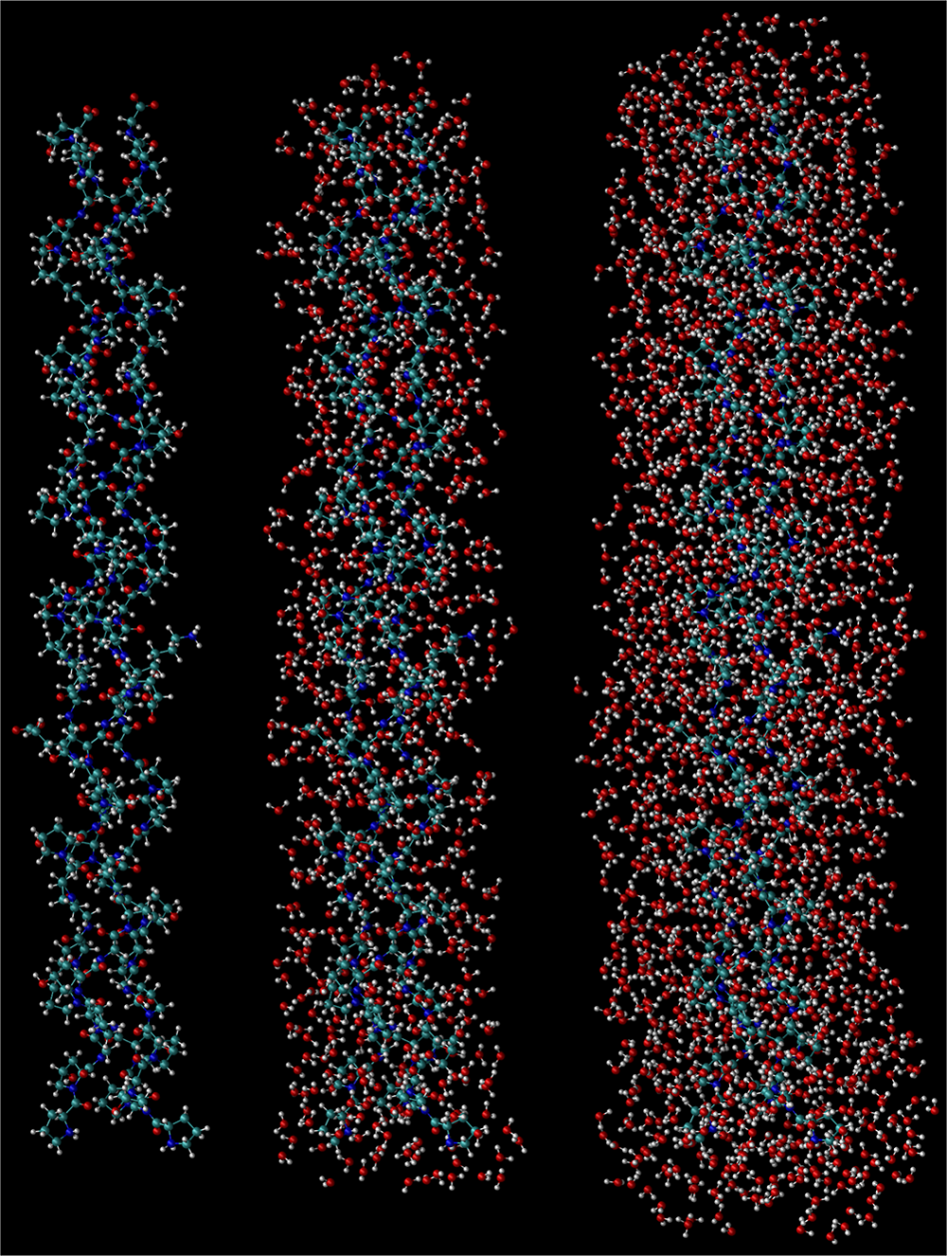
Snapshots from simulation illustrating the water selections described in section 4.1. Left: the collagen-like peptide. Middle: the peptide with the first hydration layer. Right: the peptide with the first and second hydration layers.

The analysis of relaxation in the vicinity of the peptide is separated into contributions from water in different geometrical regions around the peptide, as well as contributions from different types of coupling. Intra- and intermolecular couplings in water are referred to as *intra* and *inter*, respectively. The couplings between hydrogen nuclei in water and in the peptide are denoted with *pept. H*. The sum of *intra, inter*, and *pept.* is referred to as *all*. The different contributions are additive, provided that the cross-correlations between the corresponding dipolar couplings are negligible. Water molecules which have any atom of the collagen-like peptide closer than 3.5 Å are denoted with *1st h.l.* (*h.l.* = hydration layer) (Figure 2). Similarly, *2nd h.l.* contains the water molecules which have any atom of the peptide at a distance *r*, *r* < 7.0 Å but not at *r* < 3.5 Å. Finally, *water* contains all water molecules in the simulation with the peptide, for example, *water pept. H* refers to ^1^H–^1^H couplings between all water molecules and the peptide. The criterion for the hydration layer, *r* < 3.5 Å, contains the first two peaks of the water–peptide O–H and H–O radial distribution functions (RDFs), as well as the first peaks of the O–N and O–O RDFs (Figure S1, in the SI). Our definition for the second hydration layer is chosen on RDFs to fully contain the second hydration layer at the cost of also containing contributions of higher hydration layers. The definition of the hydration layers is rather coarse (see section S6 in the SI) but adequate for the purpose of seeing the effect of the vicinity of the peptide to the molecular motion of water. The results labeled with *bulk* are computed from the bulk water simulation, including all water molecules therein.

Spherical cutoff radii of 10 and 20 Å were used for the intermolecular couplings in the bulk and peptide simulations, respectively. In the system with the peptide, the simulation cell is anisotropic. Computing the intermolecular couplings between water molecules without cutoff would bias the RDCs toward the directions in which the simulation cell is the longest. Compared to the RDCs, the TCFs decay quickly with distance and the long-range effects are much less of concern. Although dipolar couplings decay with distance as *r*^–3^, their sum does not. So, as opposed to the correlation functions, the RDCs are long-ranged and we can not estimate them reliably from the simulation except in the case of intramolecular couplings. This limits the use of eq 14 for our analysis.

By separately studying the relaxation rates of protons in water molecules at different locations relative to the peptide, we aim to see how the vicinity of the macromolecule affects the water proton relaxation in a fibrillar environment such as articular cartilage. Average distance between collagen type II monomers in a fibril is 13 Å,^38^ which means the water inside fibrils is confined very close to the macromolecules until it eventually exchanges with the bulk water outside. The space accessible for water molecules inside the fibrils corresponds roughly to the combined first and second hydration layers in our study.

### 4.2 Analysis Based on Initial Location of Water Molecules

Two types of analysis was employed for the computation of the TCFs. In the first one, which we later refer to as “unrestricted” analysis, the trajectory given by the simulations was sliced into 100 ps blocks. The correlation functions in eq 12 were computed in the average PAS of the inertia tensor of the peptide in each of these blocks. The water selections used in this type of analysis are static and defined based on the location of the water molecule at the beginning of the 100 ps trajectory segment. This simplified definition of hydration layers allows for a more thorough statistical analysis of the results (i.e., bootstrapping) compared to the more stringent definition used in the restricted analysis below. Also, together with the restricted analysis, it can give an assessment of the impact of residence times in the hydration layers on TCFs. In practice the correlation function in this case becomes
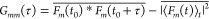
22where *t*_0_ is the beginning of the block and the latter term represents the time-independent baseline.

The water molecules in the hydration layers of the peptide eventually escape the space corresponding to their assignment. During the first 100 ps, more than 90% of the water molecules initially in the first hydration layer have left it at least once and after the first nanosecond all water molecules have left the first hydration layer. Since we compute the correlation functions as averages over 100 ps blocks, a water molecule initially in the first hydration layer has most likely spent time in the second hydration layer and possibly beyond. For these reasons, the baselines  of the correlation functions in this type of analysis are always computed for all water molecules. For the same reasons the secular RDCs in Δ were computed for all water molecules. The starting point for the TCF calculation, *t*_0_, is not iterated through the 100 ps block. Therefore, the TCF is essentially independent of block length. In section 5.2 it is seen that the correlation times of the dipole–dipole couplings computed using the trajectory segments are mostly much shorter than the length of the segment and the error due to segmenting should be relatively small.

Where shown, the confidence in the computed correlation times and relaxation rates, etc. was evaluated by bootstrapping.^39^ The 100 ps trajectory segments used in the analysis were taken as independent, giving 150 segments in the 1QSU system. From each segment, correlation functions and RDCs were computed. This combination of quantities computed from one segment was treated as a sample. Out of all samples, 150 were randomly selected with replacement, and the correlation functions and RDCs of these samples were averaged to create a bootstrap average. This resampling scheme was repeated 1000 times to give an equal number of bootstrap averages. The desired quantities, such as spectral densities or relaxation rates at specific β were computed for each of the 1000 averages. The 2.5th and 97.5th percentile of the distribution of the bootstrap averages were used as the limits of the 95% CI. The mean of the distribution is reported as the mean value of the desired quantity. We acknowledge that the 100 ps trajectory segments are not completely independent, that is, they are correlated due to the much longer time scale of the peptide motions as compared to water molecule motions. Bootstrapping was not used to compute the PAS of the inertia tensor.

### 4.3 Analysis Restricted to the First Hydration Layer

To indirectly study the effect of water molecules escaping the first hydration layer we performed also a more limited analysis in which the trajectory was used as a whole and the correlation functions were computed only during the times the water molecule stays in the first hydration layer. Results from this ”restricted” analysis have the additional label *restr.* Here, the correlation functions were computed as
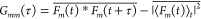
23where both *t* and *t* + τ iterate through the entire trajectory, but only those intervals [*t*, *t* + τ] during which the molecule stays in the first hydration layer were included in the analysis.

The procedure has the effect of giving a higher weight to those water molecules which stay longer times in the hydration layer. We can consider the analyses restricted to the first hydration layer and the unrestricted analysis described in section 4.2 as two limiting cases in what can be observed with our model. Error estimates are not given for the quantities computed from the restricted analysis, since the bootstrapping methodology used for the trajectory segments could not be applied in the case when the trajectory was used as a whole.

## 5 Results

### 5.1 Residual Dipolar Couplings

If one looks at the intramolecular RDC only and restricts the analysis to the first hydration layer so that the hydration layer is computed for each snapshot separately, we get an estimate for the RDC of water molecules that would stay close to the peptide. In this case the secular dipolar coupling is *d*_IS_ = 2.66 kHz. Similar computation for the second hydration layer yields 347 Hz and for all water molecules 85 Hz. From these figures, it is evident that the RDC decays fast when moving away from the peptide, but due to the fairly strong coupling close to the peptide, the averaged interaction is non-negligible even when all water molecules are taken into account.

### 5.2 Correlation Times of Dipole–Dipole Couplings

Integrated correlation times [eq 21] of dipole–dipole couplings were computed from the simulations with the two different analysis methods: the unrestricted (section 4.2), in which the molecules can escape the volume of their initial assignment (e.g., first hydration layer) and the restricted (section 4.3), in which the molecules were considered in the analysis only during the time they stay in the volume of their initial assignment. Table 1 shows that the integrated correlation times [eq 21] for intra- and intermolecular ^1^H–^1^H couplings between water hydrogen nuclei in the unrestricted analysis are always below 10 ps. The ^1^H–^1^H couplings between water and the peptide have longer correlation times, up to 36 ps. The relatively long correlation times of the water–peptide couplings also raise the τ_*m*_^′^ of the combined couplings in the first hydration layer (1st h.l. all) close to 10 ps. The τ_*m*_^′^ are mostly independent of *m*, which implies isotropic relative motion of the nuclei, except in the first hydration layer, where τ_2_^′^ = 9.7_–0.5_^+0.9^ ps is slightly longer than τ_0_^′^ = 8.1 ± 0.4 ps and τ_1_^′^ = 7.9 ± 0.3 ps for the intramolecular couplings in water. For the intermolecular couplings between hydrogen nuclei in water and the peptide τ_2_^′^ = 27.0_–0.5_^+0.6^ ps is shorter than τ_0_^′^ = 31.3_–0.7_^+0.9^ ps and τ_1_^′^ = 32.5_–0.8_^+0.9^ ps. For the couplings between hydrogen nuclei in all water molecules with the hydrogen nuclei in the peptide τ_1_^′^ = 34.8_–0.8_^+1.1^ ps is longer than τ_0_^′^ = 30.0_–0.5_^+0.6^ ps and τ_2_^′^ = 29.9 ± 0.6 ps. The combined effect of the different couplings in the first hydration layer is that τ_0_^′^ and τ_1_^′^ are the same (9.4 ± 0.3 ps) and τ_2_^′^ = 10.1_–0.3_^+0.4^ is slightly longer.

**Table 1:** Integrated Correlation Times τ_*m*_^′^ [eq 21] of ^1^H–^1^H Dipole Couplings in the Principal Axis System of the Peptide Inertia Tensor^a^

	τ_0_^′^ (ps)	τ_1_^′^ (ps)	τ_2_^′^ (ps)
1st h.l. all	9.4 ± 0.3	9.4 ± 0.3	10.1_–0.3_^+0.4^
1st h.l., intra	8.1 ± 0.4	7.9 ± 0.3	9.7_–0.5_^+0.9^
1st h.l., inter	7.50 ± 0.08	7.68 ± 0.08	7.66 ± 0.07
1st h.l., pept. H	31.3_–0.7_^+0.9^	32.5_–0.8_^+0.9^	27.0_–0.5_^+0.6^
2nd h.l. all	3.91_–0.10_^+0.11^	3.96 ± 0.08	4.01 ± 0.06
2nd h.l., intra	2.97_–0.1_^+0.13^	3.01_–0.07_^+0.08^	3.06_–0.08_^+0.09^
2nd h.l., inter	5.01 ± 0.04	5.09 ± 0.03	5.10 ± 0.03
2nd h.l., pept. H	32 ± 2	36 ± 2	30.3_–0.9_^+1.2^
water intra	2.58 ± 0.02	2.59 ± 0.02	2.61 ± 0.02
water inter	4.03 ± 0.01	4.034_–0.009_^+0.010^	4.038_–0.009_^+0.010^
water, pept. H	30.0_–0.5_^+0.6^	34.8_–0.8_^+1.1^	29.9 ± 0.6
bulk intra	2.45 ± 0.04	2.46 ± 0.04	2.49_–0.03_^+0.04^
bulk inter	3.57 ± 0.03	3.58_–0.03_^+0.02^	3.57 ± 0.03
1st h.l., intra, restr.	54.8	39.3	150
1st h.l., inter, restr.	16.8	17.2	17.2
1st h.l., pept. H, restr.	468	462	377

a The errors represent 95% confidence intervals computed with the bootstrap method. See section 4.1 for definitions of the water selections.

When the analysis of the couplings is restricted strictly to the water molecules in the first hydration layer of the peptide and thus the effect of water molecules escaping the vicinity of the peptide is eliminated, the correlation times for intramolecular couplings are much longer and range from τ_1_^′^ = 39.3 ps to τ_2_^′^ = 150 ps (Table 1). Remarkably, τ_2_^′^ is much longer than τ_0_^′^ and τ_1_^′^. The intermolecular couplings between water are less affected and the τ_*m*_^′^ with different *m* are very close to each other at around 17 ps. The correlation times for the water–peptide couplings are the longest ranging from τ_2_^′^ = 377 ps to τ_0_^′^ = 468 ps, roughly an order of magnitude longer than when only the initial position of the water molecules was considered in the analysis. Similarly to the nonrestricted analysis, τ_2_^′^ is shorter than τ_0_^′^ or τ_1_^′^ for the water–peptide couplings.

### 5.3 Spectral Densities

**Figure 3:**
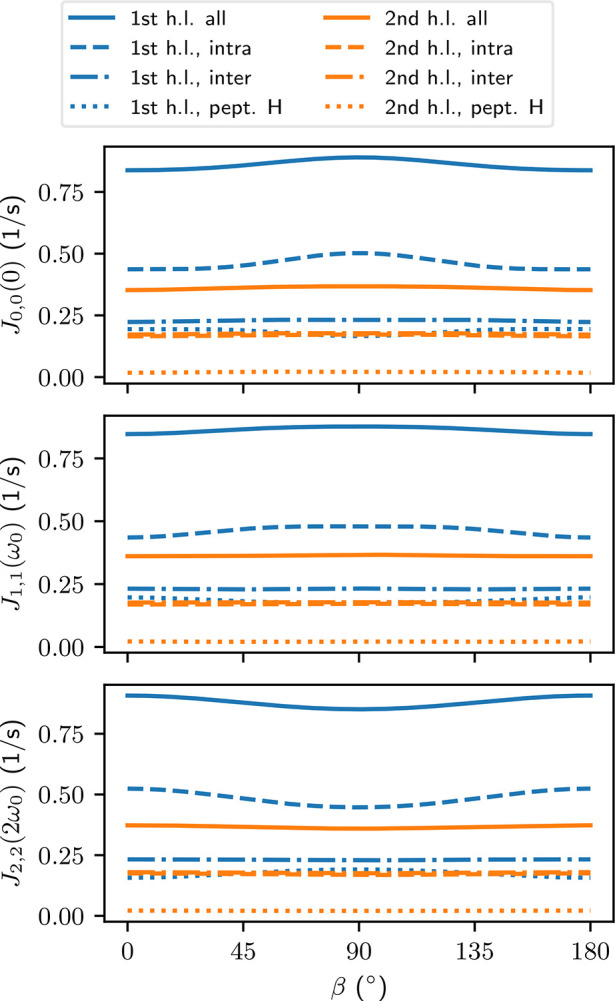
Spectral densities (ω_0_ = 2π × 400 MHz) as a function of the angle β between the peptide and the main magnetic field. Each curve represents a different kind of water selection and coupling. See section 4.1 for an explanation of the labels.

Spectral densities [eq 20] at  MHz were computed from the fitted correlation functions as explained in section 4. The Larmor frequency used corresponds to field strength *B*_0_ = 9.4 T. The spectral densities show how the different molecular motions transfer to the orientation-dependency of the relaxation rates. From Figure 3 we see that for most of the water selections, the spectral densities are relatively independent of the peptide orientation. The magnitudes of *J*_*m*,*m*_ with different *m* are also close to each other for the same contribution. These findings are confirmed in Tables S1 and S2 of section S8 in the SI, where we take a more detailed look at the spectral densities. The notable exception to the mostly isotropic behavior are the *J*_*m*,*m*_ of the intramolecular couplings in water in the first hydration layer of the peptide (Figure 3). Here, each *J*_*m*,*m*_ with different *m* has a different orientation-dependency. The *J*_0,0_(0) and *J*_1,1_(ω_0_) have maxima at β = 90°, whereas *J*_2,2_(2ω_0_) has a minimum at the same orientation. Since all spectral densities contribute to the relaxation rates with a positive sign, the anisotropies partially cancel each other.

### 5.4 Longitudinal Relaxation

Longitudinal relaxation rates *R*_1_ at ν_0_ = 400 MHz were computed from the simulated trajectory using equation 9 and the methods presented in section 4. The results are listed in Table 2, from which one can see that the fastest individual relaxation rate, *R*_1_ = 0.63 ± 0.02 s^–1^ is found for the intramolecular couplings in water in the first hydration layer of the peptide. The total relaxation rate in the first hydration layer due to inter- and intramolecular dipolar couplings in water, as well as intermolecular couplings between water and the peptide is *R*_1_ = 1.12 ± 0.02 s^–1^. The intramolecular couplings in water account for 56% of the *R*_1_ in the first hydration layer. The intermolecular couplings in water, as well as between water and the peptide, contribute the remaining 26% and 18%, respectively. In the second hydration layer, the *R*_1_ values due to inter- and intramolecular couplings of water are almost equal to each other and the contribution from the water–peptide couplings is only 6% from the total *R*_1_ of 0.463 s^–1^. When all water molecules are taken into account the total relaxation rate is *R*_1_ = 0.322 s^–1^. The intra- and intermolecular couplings in water contribute 57.3% and 40.5% to this and the water–peptide couplings provide the remaining 2.2%.

**Table 2:** Relaxation Rates and the Fractions of Their Anisotropic Parts *g*(*R*_*i*_) = 1 – *R*_*i*,min_/*R*_*i*,max_ Computed at  MHz^a^

	*R*_1_ (1/s)	*R*_2_ (1/s)	*g*(*R*_1_)	*g*(*R*_2_)
1st h.l. all	1.12 ± 0.02	1.07 ± 0.02	0.04 ± 0.01	0.022 ± 0.006
1st h.l., intra	0.63 ± 0.02	0.57 ± 0.01	0.10 ± 0.02	0.06 ± 0.01
1st h.l., inter	0.291 ± 0.002	0.287 ± 0.002	0.013 ± 0.003	0.007 ± 0.001
1st h.l., pept. H	0.206 ± 0.002	0.235 ± 0.003	0.124_–0.005_^+0.006^	0.062 ± 0.003
2nd h.l. all	0.463_–0.006_^+0.005^	0.451 ± 0.005	0.03 ± 0.01	0.013 ± 0.006
2nd h.l., intra	0.217 ± 0.004	0.211_–0.003_^+0.004^	0.03 ± 0.02	0.014 ± 0.009
2nd h.l., inter	0.224 ± 0.001	0.220 ± 0.001	0.018 ± 0.002	0.009 ± 0.001
2nd h.l., pept. H	0.0273_–0.0003_^+0.0004^	0.0257_–0.0005_^+0.0007^	0.06_–0.02_^+0.01^	0.030_–0.009_^+0.007^
water intra	0.1846 ± 0.0010	0.1836 ± 0.0008	0.005_–0.003_^+0.004^	0.003 ± 0.002
water inter	0.1304 ± 0.0003	0.1306 ± 0.0003	0.0013 ± 0.0006	0.0006 ± 0.0003
water, pept. H	0.00714 ± 0.00007	0.00763 ± 0.00009	0.063 ± 0.005	0.032 ± 0.002
bulk intra	0.176 ± 0.002	0.175 ± 0.002	0.007_–0.008_^+0.013^	0.003_–0.004_^+0.006^
bulk inter	0.1160 ± 0.0008	0.1161 ± 0.0008	0.001 ± 0.002	0.0004_–0.001_^+0.0009^
1st h.l., intra, restr.	1.73	2.40	0.28	0.14
1st h.l., inter, restr.	0.611	0.617	0.0096	0.0048
1st h.l., pept. H, restr.	0.637	1.95	0.67	0.34

a For the peptide system, *R*_1_ and *R*_2_ were computed in the PAS of the inertia tensor (β = 0). For the bulk system, axes of the coordinate system were aligned along the vertices of the simulation box. The *g*(*R*_*i*_) were computed from the minimum and maximum values of *R*_*i*_ with respect to the angle β between the long axis of the peptide and **B**_0_. The errors represent 95% confidence intervals computed with the bootstrap method. See section 4.1 for an explanation of the labels.

**Figure 4:**
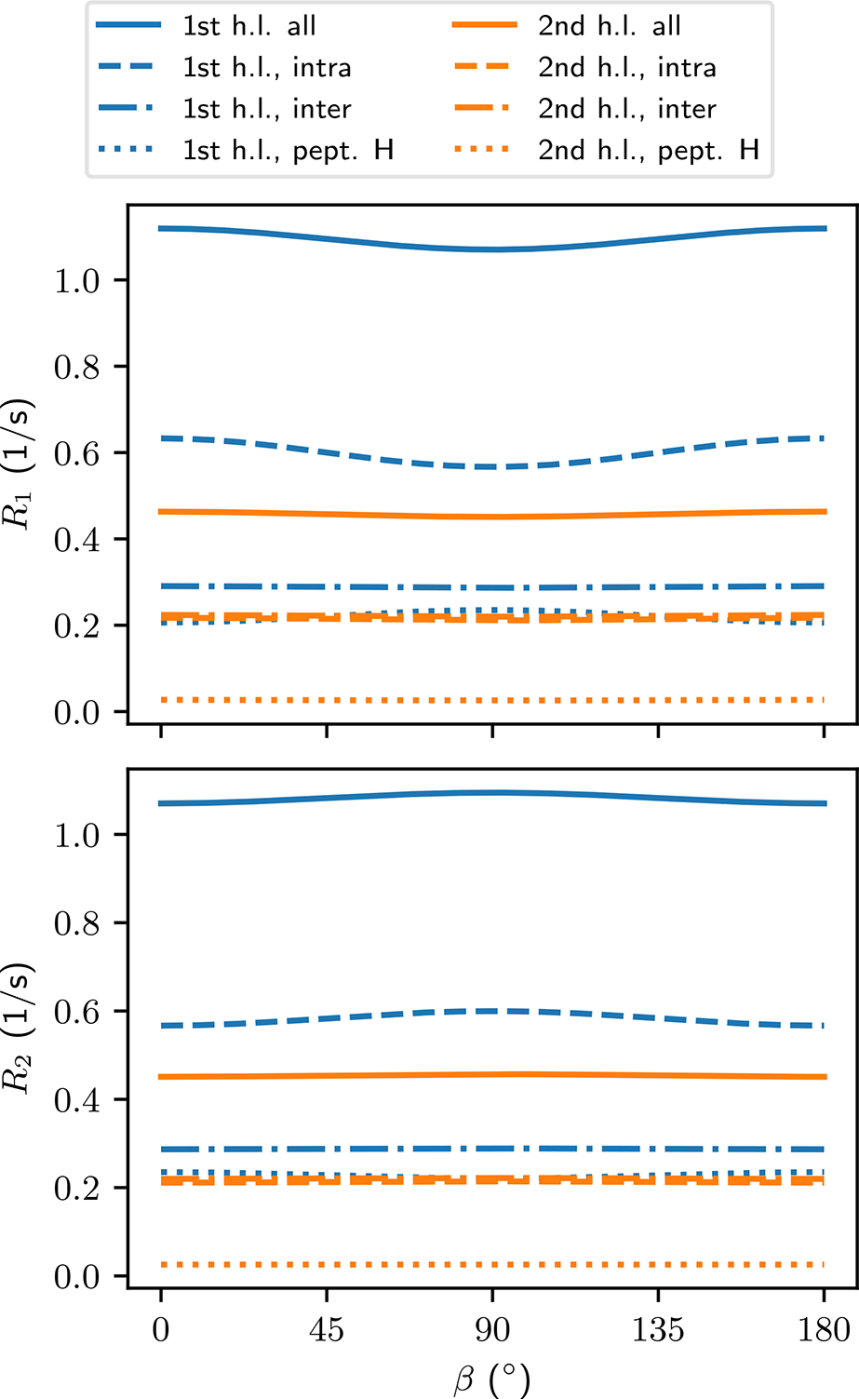
Longitudinal and transverse relaxation rates *R*_1_ and *R*_2_ [eqs 9 and 18] of water protons due to ^1^H–^1^H dipole–dipole couplings as a function of the angle β between the peptide and the main magnetic field (see Figure 1). Each curve represents a different kind of water selection and coupling. See section 4.1 for an explanation of the labels.

From Figure 4 we see that most of the relaxation rates are isotropic. The notable exception is the *R*_1_ for intramolecular dipole–dipole couplings for the water molecules in the first hydration layer of the peptide. There, a clear minimum is observed when the peptide is oriented perpendicular to *B*_0_. From the spectral densities in Figure 3 we can see that this minimum is mostly due to *J*_2,2_(2ω_0_). The anisotropic part of *R*_1_ is 10 ± 2% of the whole relaxation rate (Table 2). Already in the second hydration layer this anisotropy vanishes (drops below 2%) and becomes insignificant when all water molecules are taken in to account.

### 5.5 Evolution of the Transverse Magnetization

Table 2 shows that *R*_2_ behaves mostly similar to *R*_1_ and therefore we will not repeat all the findings made in section 5.4. The values of the longitudinal and transverse relaxation rates differ mainly in the intramolecular couplings of water in the first hydration layer, where *R*_2_ = 0.57 ± 0.01 s^–1^ is slightly slower than *R*_1_ = 0.63 ± 0.02 s^–1^. This difference is translated to the overall relaxation rates in the first hydration layer. Small differences between the two relaxation rates appear also in the water–peptide couplings, but their overall effect in the relaxation is small. Notably, *R*_2_ for the combined couplings in the first hydration layer is less anisotropic than *R*_1_ (Table 2), and any anisotropy in the former is masked by the errors resulting from fitting the TCFs.

**Figure 5:**
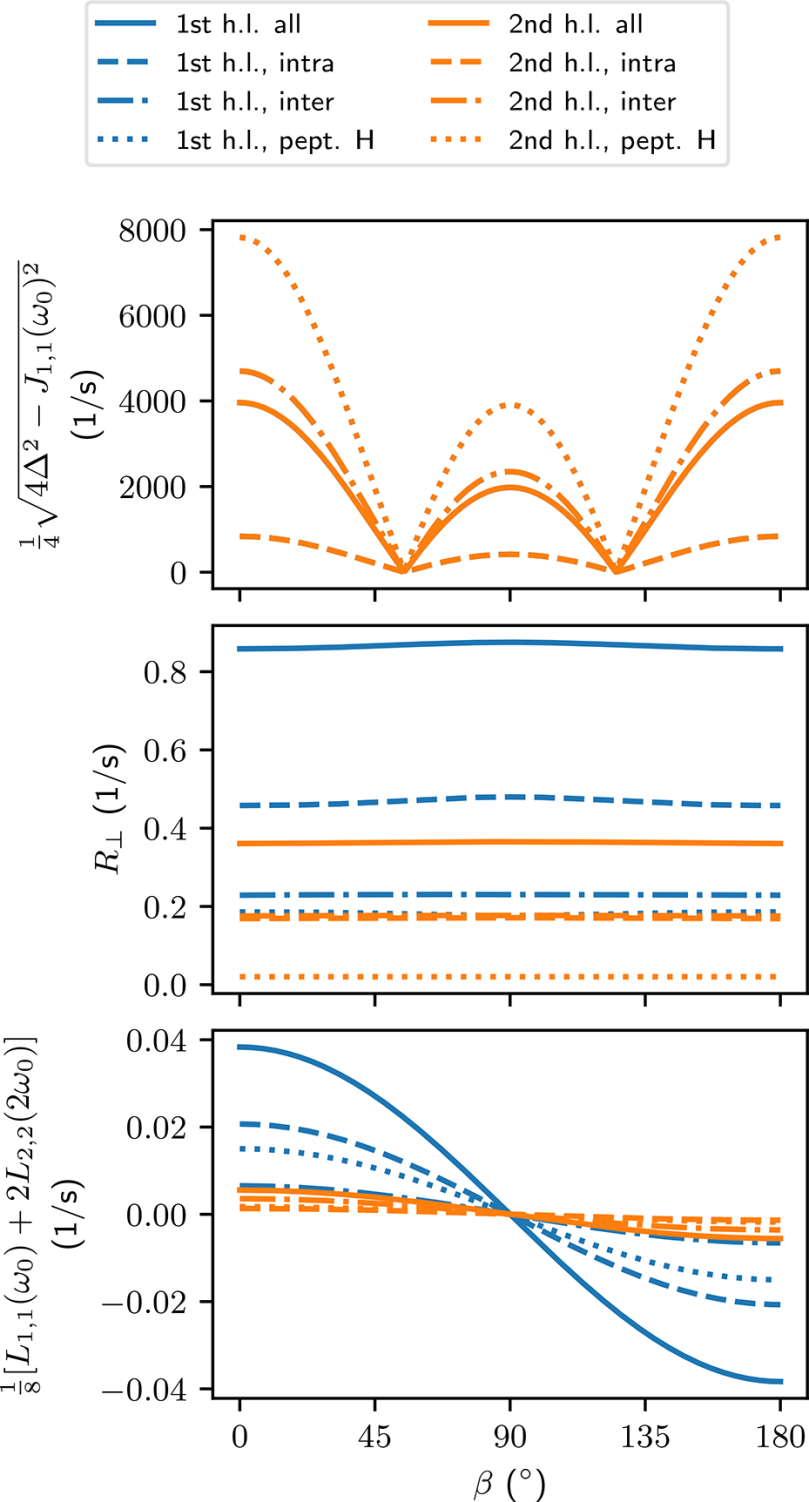
Components affecting the time-evolution of transverse magnetization [eqs 14 and 17] as a function of the angle β between the long axis of the peptide and the main magnetic field. Each curve represents a different kind of water selection and coupling. See section 4.1 for an explanation of the labels.

RDCs were computed for all water molecules, similarly as in the case of baselines of the TCFs (section 4.1), and components of eq 14 were evaluated individually (Table 3). The decay rate, *R*_⊥_ [eq 17] in Figure 5 is almost invariable with orientation, except for the intramolecular couplings in the first hydration layer, where the rate has a weak maximum at 90°. Overall, *R*_⊥_ is isotropic in the first hydration layer.

**Table 3:** Components of the Evolution of the Transverse Magnetization at  MHz^a^

	*R*_⊥_ (1/s)	^1^/_4_(4Δ^2^ – *J*_1,1_ (ω_0_)^2^)^1/2^ (1000/s)	^1^/_8_[*L*_1,1_(ω_0_) + 2*L*_2,2_(2ω_0_)] (1/s)
1st h.l. all	0.86 ± 0.01		0.038 ± 0.003
1st h.l., intra	0.46 ± 0.01		0.021_–0.003_^+0.004^
1st h.l., inter	0.229 ± 0.002		0.0065 ± 0.0001
1st h.l., pept. H	0.186 ± 0.002		0.0150 ± 0.0005
2nd h.l. all	0.361 ± 0.004		0.0056 ± 0.0004
2nd h.l., intra	0.169_–0.002_^+0.003^		0.0013_–0.0001_^+0.0002^
2nd h.l., inter	0.1757_–0.0010_^+0.0009^		0.00358 ± 0.00005
2nd h.l., pept. H	0.0202_–0.0004_^+0.0005^		0.00176_–0.00010_^+0.00014^
water intra	0.1469 ± 0.0007	0.8 ± 0.1	0.00087 ± 0.00002
water inter	0.1044_–0.0002_^+0.0003^	4.7 ± 0.1	0.001611 ± 0.000009
water, pept. H	0.00598 ± 0.00007	7.82 ± 0.08	0.00054 ± 0.00002

a Errors represent 95% confidence intervals computed with the bootstrap method. See section 4.1 for an explanation of the labels. The oscillation factor ^1^/_4_(4Δ^2^ – *J*_1,1_ (ω_0_)^2^)^1/2^ is reported only for all water molecules, since the same secular RDC values (Δ/3) were used for the analysis of the hydration layers. See section 4.1 for discussion.

The term  defining the oscillation frequency in eq 14 is dominated by the secular RDC (Δ/3) and therefore its orientation dependency follows quite closely a |(3 cos^2^ β – 1)| form as seen in Figure 5. The term inside the square root is positive, except perhaps very close to the magic angles 54.74° and 125.26°, where RDC vanishes. Due to partial cancellation of different contributions, the RDC from all couplings combined is smaller in magnitude than the individual contributions from water–peptide or intermolecular couplings in water. Long-range contributions to the intermolecular couplings were not evaluated, and therefore any values depending on them, such as the combined couplings in Figure 5 should be taken merely as suggestive.

The other oscillating term in eq 14 has angular frequency −0.021 s^–1^ < ^1^/_8_[*L*_1,1_(ω_0_) + 2*L*_2,2_(2 ω_0_)] < 0.021 s^–1^, which is very low. This means that we can treat the corresponding cosine term in eq 14 as equal to 1, as anticipated in section 2.

### 5.6 Orientation-Dependency of Relaxation Times

Relaxation times *T*_1,2_ can be calculated from the corresponding relaxation rates *R*_1,2_. For bulk water, *T*_1_ ≈ *T*_2_ = 3.4 s. For the water with the peptide we saw in sections 5.4 and 5.5 that only the total *R*_1_ in the first hydration layer was slightly anisotropic when the uncertainties in determining the relaxation rates were taken into account. The corresponding maximum and minimum values are *T*_1_(90°) = 0.93 ± 0.01 s and *T*_1_(0°) = 0.89_–0.02_^+0.01^ s, respectively. The transverse relaxation time in the first hydration layer is *T*_2_ = 0.93 ± 0.01 s. In the second hydration layer the relaxation times agree within errors: *T*_1_ = 2.16 ± 0.03 s and *T*_2_ = 2.19 ± 0.02 s. For all water molecules in the peptide simulation, longitudinal and transverse relaxation times are equal within errors. *T*_1_ = 3.10 s, which is 10% faster than the simulated longitudinal transverse relaxation times for bulk water, *T*_1_ = 3.42 s.

### 5.7 Relaxation in the Analysis Restricted to the First Hydration Layer

**Figure 6:**
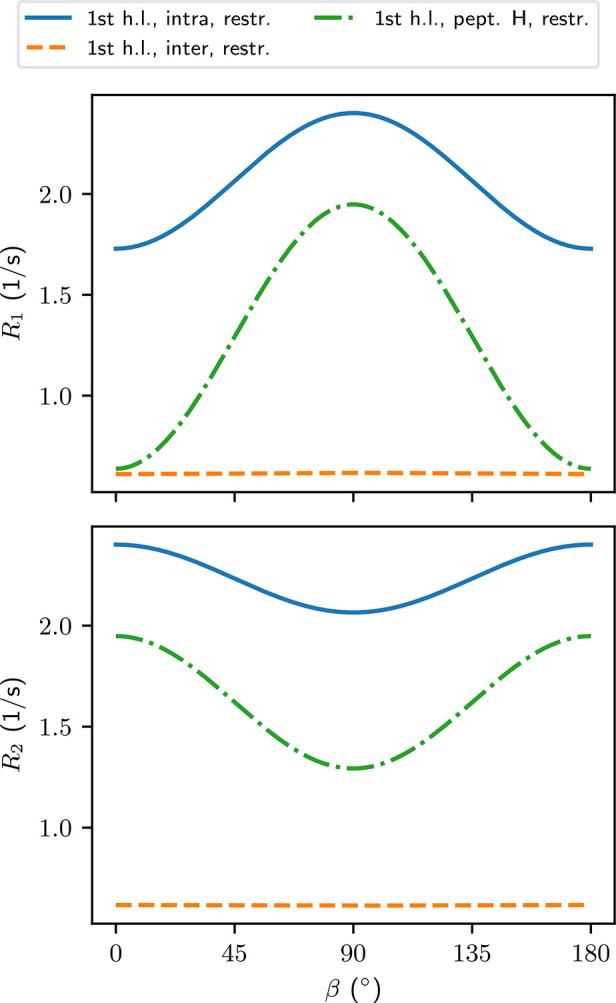
Relaxation rates computed from the time intervals during which the molecules stay in the first hydration layer. β is the angle between the long axis of the peptide and the main magnetic field.

Relaxation rates computed with the analysis restricted to the first hydration layer (section 4.3) are generally faster than in the unrestricted analysis (Table 2). The fastest relaxation rates are found for the intramolecular couplings. Here *R*_1_ and *R*_2_ share the same maximum value 2.40 s^–1^, which occurs for *R*_1_ at β = 90° and for *R*_2_ at β = 0° (Figure 6). The minimum values for the two relaxation rates are different with *R*_1_ minimum 1.73 s^–1^ appearing at β = 0° and *R*_2_ minimum 2.07 s^–1^ seen at β = 90°. The relaxation rates due to water–peptide ^1^H–^1^H couplings result in relaxation rates of slightly smaller magnitude, but stronger anisotropy (Table 2). The maximum and minimum *R*_1_ due to the water–peptide couplings are 1.95 s^–1^ and 0.637 s^–1^, at β = 90° and 0°, respectively (Figure 6). The *R*_2_ due to the water–peptide couplings is less anisotropic and the anisotropy is inverted as compared to *R*_1_. Maximum and minimum *R*_2_ are 1.95 s^–1^ and 1.29 s^–1^, at β = 0° and 90°, respectively. The relaxation rates due to intermolecular ^1^H–^1^H couplings between water molecules are, in essence, isotropic with the differences between the orientations falling below 1%. The longitudinal and transverse relaxation rates are also practically equal to each other, with *R*_1_ = *R*_2_ = 0.617 s^–1^ at their respective maxima.

**Figure 7:**
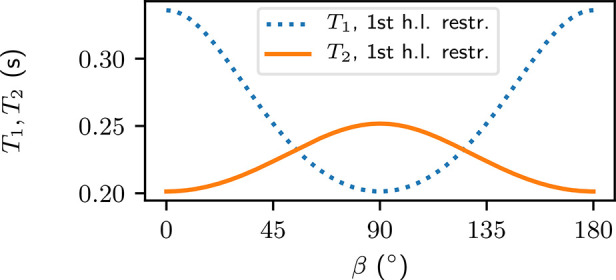
Relaxation times computed from the time intervals during which the molecules stay in the first hydration layer. β is the angle between the long axis of the peptide and the main magnetic field.

The relaxation times computed by summing up the relaxation rates from the restricted analysis give both *T*_1_ and *T*_2_ a minimum of 0.201 s (Figure 7). The shortest *T*_1_ appears at β = 90°, whereas the shortest *T*_2_ is seen at β = 0°. The maxima of the two relaxation rates appear at orientations with 90-degree differences to the minima. At their respective maxima, *T*_1_ = 0.336 s is 1.3 times longer than *T*_2_ = 0.252 s.

## 6 Discussion

We have computed relaxation of water hydrogen nuclei due to ^1^H–^1^H dipolar coupling from a MD simulation of a collagen-like peptide dissolved in water. Results from a bulk water simulation were used as a reference. Redfield relaxation theory including RDC was derived for a pair of spin-^1^/_2_ nuclei (section 2 and SI sections S1–S5). In the system with the peptide, our two analysis strategies, the unrestricted and the restricted, could be considered as estimates of the opposite extremes. In the former, the water is fairly free to self-diffuse, and in the latter case it is confined to the macromolecular structure.

Only weak anisotropy was found in the relaxation times computed with the unrestricted analysis. The correlation times of the dipole–dipole couplings between water hydrogens were below 10 ps even in the first hydration layer. The correlation times of the couplings between hydrogens in water and the peptide were slightly longer, but still below 40 ps. The relaxation times *T*_1_ and *T*_2_ were 0.9 s in the first hydration layer and approached bulk water relaxation times further away from the peptide. In the analysis restricted to the first hydration layer, the simulated correlation times of the intramolecular dipole–dipole couplings varied from τ_1_^′^ = 39.3 ps to τ_2_^′^ = 150 ps (Table 1), reflecting significant anisotropy. For the water–peptide ^1^H–^1^H couplings, the longest correlation time was on the order of 470 ps, roughly 2 orders of magnitude longer than in bulk water. The resulting relaxation times varied from 201 to 336 ms, depending on peptide orientation.

The anisotropy in the correlation times can be analyzed by looking at the spherical harmonics in Cartesian coordinates [eq 8]. The *z*-axis corresponds to the vector along the long axis of the peptide (Figure 1) with the *x*, *y*-plane perpendicular to this vector. For the intramolecular couplings in the first hydration layer, τ_1_^′^ as the shortest correlation time would fit together with a diffusion in a cone model, in which one of the hydrogen atoms in the water molecule is bonded to the peptide via a hydrogen bond and the other hydrogen is moving more freely. This would make the mixed motion, involving movement along the *z*′ axis as well as in the *x*′, *y*′ plane, the fastest out of the three options. The anisotropy in the intermolecular couplings with the peptide is harder to interpret, as translational motion is also included. However, one could relate the shortest correlation time τ_2_^′^ to the fact that for a water molecule adjacent to the peptide, the *x*, *y*-plane provides a way out of the peptide’s proximity to diffuse into the bulk water.

### 6.1 Comparison of the Unrestricted and Restricted Analysis

The relaxation rates computed from the restricted analysis are generally faster than in the unrestricted analysis. The differences in the relaxation rates between the two analysis methods are the smallest in the intermolecular couplings between water hydrogens. Even there, the restricted analysis results in *R*_1_ and *R*_2_ twice as fast as in the unrestricted analysis. For intramolecular couplings, the relaxation rates are three to four times faster than in the unrestricted analysis, in which only the initial positions of the water molecules were taken into account. The most dramatic difference is seen in the relaxation rates resulting from the water–peptide ^1^H–^1^H couplings, where *R*_2_ is almost an order of magnitude faster in the restricted analysis as compared to the unrestricted (Table 2). The anisotropy of the relaxation rates in the restricted analysis is 2–3 times higher for the intramolecular couplings and 5 times higher for the couplings between water and peptide as compared to the corresponding anisotropies in the nonrestricted analysis (Table 2).

The relaxation times *T*_1_ and *T*_2_ computed from the restricted analysis (Figure 7) are up to 78% shorter as compared to the unrestricted analysis (section 5.2). In the restricted analysis the anisotropic part makes up 40% of *T*_1_, while in the unrestricted analysis it is only 4%. The anisotropy in *T*_2_ was considered negligible in the unrestricted analysis, while in the restricted analysis the anisotropic part of *T*_2_ contributes up to 20% of the relaxation time.

When the relaxation rates were computed by taking into account the water molecules only while they stay at the first hydration layer (Figure 6), a striking difference to the case of nonrestricted analysis (Figure 4) is seen: the anisotropies of the relaxation rates due to intramolecular couplings are not just different in magnitude but have different sign. This is likely due to the higher weighting given implicitly to the molecules which stay longer in the hydration layer.

The orientation dependence of *T*_1_ (Figure 7) is inverted as compared to what was found for the unrestricted analysis (section 5.6) in the first hydration layer. This is due to the strong anisotropic contribution from the relaxation rate related to couplings between water and the peptide (Figure 6) and the inverted anisotropy of the relaxation rates related to intramolecular couplings in water. However, not all of the TCFs of the water–peptide couplings were the shape of a multiexponential, which made the TCF fits less accurate than in the case of the analysis based only on the initial positions of the water molecules.

### 6.2 Comparison to Previous Experiments and Simulations

The correlation times of intramolecular couplings are directly the rotational correlation times of the H–H vector in a rigid water molecule. The 2.45 ps correlation times obtained for the bulk water correspond well to reorientation times in other simulation works at similar temperatures and pressures.^23,24,40^ The correlation times of intramolecular couplings in the first hydration layer in the unrestricted analysis are roughly 3 times longer than the bulk water intramolecular correlation times. This translates to a rather moderate slowdown of water reorientation in the vicinity of the peptide.^23^ In the restricted analysis, strikingly longer correlation times of intramolecular couplings, ranging from 39.3 to 150 ps were observed. However, this analysis gives more weight to those water molecules that stay longer times close to the peptide.

Earlier, by analyzing the residence times of water molecules in different hydrogen-bonding conditions, we found that most of the slowdown appears to result from cases in which the water molecules act as donors in a hydrogen bond.^41^ The residence times and hydrogen-bonding has been analyzed for a similar collagen-like peptide more thoroughly earlier by Tourell and Momot.^17^

Our *T*_1_ = 3.42 s computed from the bulk water simulation is slightly shorter than the measured *T*_1_ for pure water, which ranges from 3.57 s (25 °C)^42^ to *T*_1_ = 3.838 s (27.50 °C).^43^ Previous molecular dynamics simulations using the same water model have given the value *T*_1_ = 3.8 s at 298 K.^24^ We were not able to trace the source of the difference to our result, although bulk simulations in different ensembles as well as removing the cutoff from the analysis of the bulk simulation were tested.

The correlation times computed from our simulations with the unrestricted analysis range from 2.45 to 36 ps (Table 1) so ω_0_τ_c_ ≪ 1 and both *R*_1_ and *R*_2_ are to an excellent approximation independent of ω_0_, when ω_0_ is below 10^9^ 1/s. Even in the case of the longest simulated correlation time from the restricted analysis, τ = 468 ps, the relaxation rates (and times) are independent of ω_0_ = 2πν_0_, when ν_0_ ≪ 340 MHz. Experimentally it has been found that *T*_1_ in collagen solutions increases with the Larmor frequency of the proton, ν_0_, in the range ν_0_ = 4.5, ···, 62 MHz.^44,45^ This suggests that dipolar relaxation, at least as described with our simple model, is not the main source of longitudinal relaxation in articular cartilage. *T*_1_ is also generally thought to be isotropic or weakly anisotropic in cartilage^4−7^ and weakly anisotropic in tendon.^4,8^ We found that *T*_1_ was generally slightly anisotropic in the first hydration layer of the peptide but in practice this effect may be hard or impossible to observe in tissues such as articular cartilage, with an imperfect alignment of collagen fibers. When computation of the TCFs was strictly restricted to the molecules staying in the first hydration layer, the anisotropy of relaxation times was amplified and reversed due to the implicit stronger weighting of the molecules with longer residence times. In the case of the restricted analysis, the transverse relaxation time in the first hydration layer approaches those determined for the ”bulk” water fraction in a solution with high (37 wt %) collagen concentration.^46^ In our view, this water fraction would be better described as “fast-exchanging”, since all of the water has access to the collagen surface and forms short-lived hydrogen-bound states.

The transverse relaxation times in articular cartilage are typically in the range of tens of milliseconds when  MHz,^7^ whereas even the shortest *T*_2_ obtained in the restricted analysis of the first hydration layer in the current work was longer than 200 ms. If intramolecular couplings in water would be the dominating contributor to relaxation, according to eq 18, the experimentally observed relaxation times would correspond to correlation times in the subnanosecond (10^–10^ s) range. Compared to the bulk water rotational correlation times, this would represent a slowdown of 2 orders of magnitude. In our work, only a moderate slowdown of the rotational correlation time was observed.

*T*_2_ is known to be strongly anisotropic in articular cartilage and has a maximum when the collagen fibrils are at 55° angle with respect to the main magnetic field.^6,10^ Our simulated orientation-dependence of *T*_2_ (Figure 7) and the oscillation factor (Figure 5) suggest that the magic-angle effect at 55° could more likely be a dephasing effect caused by RDCs, than a result of slowdown of the reorientational motion of water. The former could be a result of anisotropic reorientation, whereas the latter is commonly attributed to water bound to the collagen as water bridges.^11,12^

### 6.3 Limitations and Outlook

We note that when the overall motion is anisotropic and residual dipolar interaction is included, the common formula [eq 18] for the transverse relaxation in liquids can only be taken as an approximation in anisotropic systems and a full treatment should include the dephasing effects of the RDC [eq 14]. Since the theory is strictly valid only for an isolated homonuclear spin-1/2 pair, the results for intermolecular couplings are estimates at best. Cross-correlations between different dipolar-coupled spin pairs were assumed negligible, but the assumption was not validated. A more severe limitation of the calculations was using a cutoff for the intermolecular couplings. This affects mostly the intermolecular RDCs. The even shorter cutoff used in the bulk simulation was not found to be a significant source of the error in the relaxation times of that system. Long-range corrections could be envisioned with, for example, Ewald summation or other methods for the RDCs.^47−50^ Self-diffusion of water should be included with a realistic time scale, possibly unattainable in atomistic simulation. A larger system of bulk water would allow a longer cutoff.

Naturally, a single, relatively short triple-helix can not capture all aspects of the collagen–water interactions. For example, the water trapped inside fibrils could have significant interactions with several tropocollagen molecules at the same time. This shortcoming of the model could possibly be overcome by constructing a (staggered) bundle of parallel peptides. The amount of water inside the fibrils should be small as compared to the total water content in the system. The reorientation and diffusion of interstitial water could be significantly different from the relatively free water dynamically bonding with the collagen-like peptide. The shortness of the 1QSU peptide as compared to a real tropocollagen could overrepresent the interactions of water with the ends of the peptide. Moreover, the strongly charged termini in the peptide could also contribute to this overestimation. This aspect could be tested by, for example, excluding water near the termini from the analysis.

The current simulations are too short to realistically describe the movement of the peptide in solution, which is much slower than the movement of water molecules. Anyway, we are more interested in the cases in which the water molecules interact with a collagen strand that preserves its orientation, at least on average. The difference in the time scales of the water and peptide motions justifies separating them in the analysis.

Unfortunately, the simple peptide model can not accurately reproduce dephasing effects. A realistic description of water movement in an anisotropic collagen network would be needed to address these. Attempts in this direction have been carried out by others.^20^

Altogether our work presents a first step toward modeling dipolar relaxation of water hydrogens in a guest–host system in which a collagen matrix acts as a host and water molecules as a guest. More realistic fibril models could be created by wrapping a tropocollagen model into a unit cell.^51,52^ This would require a complete type-II tropocollagen molecule instead of the fragment-like peptide employed here. Approaching experimental conditions could be made by including effects of water diffusion inside and outside the fibrils as well as exchange with the bulk. Information on the orientation distribution of collagen fibrils in a tissue could be obtained, for example, via polarized light microscopy.^53^

## 7 Conclusions

We found that in the first hydration layer of the peptide, the longitudinal relaxation time *T*_1_ is weakly anisotropic with extrema found when the long axis of the peptide is oriented either perpendicular or parallel to the main magnetic field. The source of this anisotropy is mainly the intramolecular dipole–dipole couplings, with water–peptide couplings yielding weaker anisotropic contributions. Overall, *T*_2_ was found to be isotropic, although individual contributions from the intramolecular couplings in water, as well as intermolecular couplings between ^1^H in water and the peptide resulted in anisotropic relaxation rates. For water molecules further away from the peptide, both relaxation times are isotropic. When the water molecules were free to escape the hydration layers, the correlation times of the dipolar couplings were found to be mostly in the picosecond range. This makes the relaxation rates practically independent of *B*_0_. When only the molecules staying in the first hydration layer of the peptide were analyzed, the correlation times were significantly longer, but still well below one nanosecond. In this case of restricted analysis, the relaxation rates/times were found to be strongly anisotropic, with the exception of intermolecular couplings between water molecules. In light of our results the experimentally observed transverse relaxation rate in articular cartilage can not be explained by dipolar relaxation in the framework of the Redfield theory, but appears to be dominated by other relaxation mechanisms or dephasing effects resulting from RDCs.

## Supporting Information Available

Redfield relaxation theory for dipolar-coupled spin-1/2 pair with residual dipolar coupling, with cylindrically symmetric relative motion of the two nuclei; radial distribution functions between water and the peptide; justification of cylindrical symmetry; anisotropy of the simulated spectral densities (PDF)

## References

[ref1] Gottardi R.; Hansen U.; Raiteri R.; Loparic M.; Düggelin M.; Mathys D.; Friederich N. F.; Bruckner P.; Stolz M. Supramolecular Organization of Collagen Fibrils in Healthy and Osteoarthritic Human Knee and Hip Joint Cartilage. PLoS One 2016, 11, e0163552 10.1371/journal.pone.0163552. 27780246 PMC5079628

[ref2] Fox A. J. S.; Bedi A.; Rodeo S. A. The Basic Science of Articular Cartilage. Sports Health 2009, 1, 461–468. 10.1177/1941738109350438. 23015907 PMC3445147

[ref3] Saarakkala S.; Julkunen P.; Kiviranta P.; Mäkitalo J.; Jurvelin J.; Korhonen R. Depth-Wise Progression of Osteoarthritis in Human Articular Cartilage: Investigation of Composition, Structure and Biomechanics. Osteoarthr. Cartil. 2010, 18, 73–81. 10.1016/j.joca.2009.08.003. 19733642

[ref4] Henkelman R. M.; Stanisz G. J.; Kim J. K.; Bronskill M. J. Anisotropy of NMR Properties of Tissues. Magn. Reson. Med. 1994, 32, 592–601. 10.1002/mrm.1910320508. 7808260

[ref5] Xia Y. Relaxation Anisotropy in Cartilage by NMR Microscopy (μMRI) at 14-μm Resolution. Magn. Reson. Med. 1998, 39, 941–949. 10.1002/mrm.1910390612. 9621918

[ref6] Hänninen N.; Rautiainen J.; Rieppo L.; Saarakkala S.; Nissi M. J. Orientation Anisotropy of Quantitative MRI Relaxation Parameters in Ordered Tissue. Sci. Rep. 2017, 7, 9606 10.1038/s41598-017-10053-2. 28852032 PMC5574987

[ref7] Hänninen N. E.; Nykänen O.; Prakash M.; Hanni M.; Nieminen M. T.; Nissi M. J. Orientation Anisotropy of Quantitative MRI Parameters in Degenerated Human Articular Cartilage. J. Orthop. Res. 2021, 39, 861–870. 10.1002/jor.24778. 32543737

[ref8] Peto S.; Gillis P.; Henri V. P. Structure and Dynamics of Water in Tendon from NMR Relaxation Measurements. Biophys. J. 1990, 57, 71–84. 10.1016/S0006-3495(90)82508-X. 2297563 PMC1280644

[ref9] Akella S. V.; Regatte R. R.; Wheaton A. J.; Borthakur A.; Reddy R. Reduction of Residual Dipolar Interaction in Cartilage by Spin-Lock Technique. Magn. Reson. Med. 2004, 52, 1103–1109. 10.1002/mrm.20241. 15508163

[ref10] Xia Y. Magic-Angle Effect in Magnetic Resonance Imaging of Articular Cartilage: A Review. Investig. Radiolog. 2000, 35, 602 10.1097/00004424-200010000-00007. 11041155

[ref11] Fullerton G. D.; Rahal A. Collagen Structure: The Molecular Source of the Tendon Magic Angle Effect. J. Magn. Reson. Imaging 2007, 25, 345–361. 10.1002/jmri.20808. 17260393

[ref12] Momot K. I.; Pope J. M.; Wellard R. M. Anisotropy of Spin Relaxation of Water Protons in Cartilage and Tendon. NMR Biomed. 2010, 23, 313–324. 10.1002/nbm.1466. 20013798

[ref13] Furman G. B.; Goren S. D.; Meerovich V. M.; Sokolovsky V. L. Anisotropy of Spin–Spin and Spin–Lattice Relaxation Times in Liquids Entrapped in Nanocavities: Application to MRI Study of Biological Systems. J. Magn. Reson. 2016, 263, 71–78. 10.1016/j.jmr.2015.12.015. 26773529

[ref14] Furman G. B.; Meerovich V. M.; Sokolovsky V. L. Correlation of Transverse Relaxation Time with Structure of Biological Tissue. J. Magn. Reson. 2016, 270, 7–11. 10.1016/j.jmr.2016.06.018. 27380185

[ref15] Duvvuri U.; Goldberg A. D.; Kranz J. K.; Hoang L.; Reddy R.; Wehrli F. W.; Wand A. J.; Englander S. W.; Leigh J. S. Water Magnetic Relaxation Dispersion in Biological Systems: The Contribution of Proton Exchange and Implications for the Noninvasive Detection of Cartilage Degradation. Proc. Natl. Acad. Sci. U.S.A. 2001, 98, 12479–12484. 10.1073/pnas.221471898. 11606754 PMC60079

[ref16] Domene C.; Jorgensen C.; Abbasi S. W. A Perspective on Structural and Computational Work on Collagen. Phys. Chem. Chem. Phys. 2016, 18, 24802–24811. 10.1039/C6CP03403A. 27711449

[ref17] Tourell M. C.; Momot K. I. Molecular Dynamics of a Hydrated Collagen Peptide: Insights into Rotational Motion and Residence Times of Single-Water Bridges in Collagen. J. Phys. Chem. B 2016, 120, 12432–12443. 10.1021/acs.jpcb.6b08499. 27973838

[ref18] Madhavi W. A. M.; Weerasinghe S.; Fullerton G. D.; Momot K. I. Structure and Dynamics of Collagen Hydration Water from Molecular Dynamics Simulations: Implications of Temperature and Pressure. J. Phys. Chem. B 2019, 123, 4901–4914. 10.1021/acs.jpcb.9b03078. 31117617

[ref19] Momot K. I. Diffusion Tensor of Water in Model Articular Cartilage. Eur. Biophys. J. 2011, 40, 81–91. 10.1007/s00249-010-0629-4. 20972563

[ref20] Powell S. K.; Momot K. I. Langevin Dynamics Modeling of the Water Diffusion Tensor in Partially Aligned Collagen Networks. Phys. Rev. E 2012, 86, 031917 10.1103/PhysRevE.86.031917. 23030954

[ref21] Persson F.; Söderhjelm P.; Halle B. How Proteins Modify Water Dynamics. J. Chem. Phys. 2018, 148, 215103 10.1063/1.5026861. 29884055

[ref22] Fogarty A. C.; Laage D. Water Dynamics in Protein Hydration Shells: The Molecular Origins of the Dynamical Perturbation. J. Phys. Chem. B 2014, 118, 7715–7729. 10.1021/jp409805p. 24479585 PMC4103960

[ref23] Laage D.; Stirnemann G.; Sterpone F.; Rey R.; Hynes J. T. Reorientation and Allied Dynamics in Water and Aqueous Solutions. Annu. Rev. Phys. Chem. 2011, 62, 395–416. 10.1146/annurev.physchem.012809.103503. 21219140

[ref24] Calero C.; Martí J.; Guárdia E. 1H Nuclear Spin Relaxation of Liquid Water from Molecular Dynamics Simulations. J. Phys. Chem. B 2015, 119, 1966–1973. 10.1021/jp510013q. 25584483

[ref25] Abragam A. The Principles of Nuclear Magnetism; Clarendon Press, 1961.

[ref26] McConnell J. The Theory of Nuclear Magnetic Relaxation in Liquids; Cambridge University Press, 1987.

[ref27] Goldman M. Formal Theory of Spin–Lattice Relaxation. J. Magn. Reson. 2001, 149, 160–187. 10.1006/jmre.2000.2239. 11318616

[ref28] Woessner D. E. Spin Relaxation Processes in a Two-Proton System Undergoing Anisotropic Reorientation. J. Chem. Phys. 1962, 36, 1–4. 10.1063/1.1732274.

[ref29] Woessner D. Nuclear Magnetic Relaxation and Structure in Aqueous Heterogenous Systems. Mol. Phys. 1977, 34, 899–920. 10.1080/00268977700102221.

[ref30] Kruk D.; Rochowski P.; Florek-Wojciechowska M.; Sebastião P. J.; Lurie D. J.; Broche L. M. 1H Spin-Lattice NMR Relaxation in the Presence of Residual Dipolar Interactions – Dipolar Relaxation Enhancement. J. Magn. Reson. 2020, 318, 106783 10.1016/j.jmr.2020.106783. 32755749

[ref31] Levitt M. H. Spin Dynamics: Basics of Nuclear Magnetic Resonance, 2nd ed.; John Wiley & Sons: Chichester, England; Hoboken, NJ, 2008.

[ref32] Kramer R. Z.; Venugopal M. G.; Bella J.; Mayville P.; Brodsky B.; Berman H. M. PDB ID: 1QSU, Staggered Molecular Packing in Crystals of a Collagen-like Peptide with a Single Charged Pair. J. Mol. Biol. 2000, 301, 1191–1205. 10.1006/jmbi.2000.4017. 10966815

[ref33] Abascal J. L. F.; Vega C. A General Purpose Model for the Condensed Phases of Water: TIP4P/2005. J. Chem. Phys. 2005, 123, 234505 10.1063/1.2121687. 16392929

[ref34] Vega C.; Abascal J. L. F. Simulating Water with Rigid Non-Polarizable Models: A General Perspective. Phys. Chem. Chem. Phys. 2011, 13, 19663–19688. 10.1039/c1cp22168j. 21927736

[ref35] Case D. A.; Berryman J. T.; Betz R. M.; Cerutti D. S.; Cheatham T. III; Darden T. A.; Duke R. E.; Giese T. J.; Gohlke H.; Goetz A. W. AMBER 2015; University of California: San Francisco, 2015.

[ref36] Phillips J. C.; Braun R.; Wang W.; Gumbart J.; Tajkhorshid E.; Villa E.; Chipot C.; Skeel R. D.; Kalé L.; Schulten K. Scalable Molecular Dynamics with NAMD. J. Comput. Chem. 2005, 26, 1781–1802. 10.1002/jcc.20289. 16222654 PMC2486339

[ref37] Hanni M.; Lantto P.; Vaara J. Nuclear Spin Relaxation Due to Chemical Shift Anisotropy of Gas-Phase 129Xe. Phys. Chem. Chem. Phys. 2011, 13, 13704–13708. 10.1039/c1cp21322a. 21709898

[ref38] Antipova O.; Orgel J. P. R. O. In Situ D-periodic Molecular Structure of Type II Collagen. J. Biol. Chem. 2010, 285, 7087–7096. 10.1074/jbc.M109.060400. 20056598 PMC2844158

[ref39] Efron B. Computers and the Theory of Statistics: Thinking the Unthinkable. SIAM Review 1979, 21, 460–480. 10.1137/1021092.

[ref40] Yeh Y.-l.; Mou C.-Y. Orientational Relaxation Dynamics of Liquid Water Studied by Molecular Dynamics Simulation. J. Phys. Chem. B 1999, 103, 3699–3705. 10.1021/jp984584r.

[ref41] Karjalainen J.; Hanni M.; Nissi M. J.; Nieminen M. T. Simulated Reorientational Correlation Times of Collagen-Associated Water. Proc. Intl. Soc. Magn. Reson. Med. 2017, 1552.

[ref42] Krynicki K. Proton Spin-Lattice Relaxation in Pure Water between 0°C and 100°C. Physica 1966, 32, 167–178. 10.1016/0031-8914(66)90113-3.

[ref43] Hindman J. C.; Svirmickas A.; Wood M. Relaxation Processes in Water. A Study of the Proton Spin-lattice Relaxation Time. J. Chem. Phys. 1973, 59, 1517–1522. 10.1063/1.1680209.

[ref44] Fung B. M.; Witschel J.; McAmis L. L. The State of Water on Hydrated Collagen as Studied by Pulsed NMR. Biopolymers 1974, 13, 1767–1776. 10.1002/bip.1974.360130910. 4370502

[ref45] Westover C. J.; Dresden M. H. Collagen Hydration: Pulsed Nuclear Magnetic Resonance Studies of Structural Transitions. Biochim. Biophys. Acta - Protein Struct. 1974, 365, 389–399. 10.1016/0005-2795(74)90011-7. 4473208

[ref46] Traore A.; Foucat L.; Renou J. P. 1H-NMR Study of Water Dynamics in Hydrated Collagen: Transverse Relaxation-Time and Diffusion Analysis. Biopolymers 2000, 53, 476–483. 10.1002/(SICI)1097-0282(200005)53:6<476::AID-BIP4>3.0.CO;2-8. 10775063

[ref47] Cerdá J. J.; Ballenegger V.; Lenz O.; Holm C. P3M Algorithm for Dipolar Interactions. J. Chem. Phys. 2008, 129, 234104 10.1063/1.3000389. 19102523

[ref48] Wang D.; Liu J.; Zhang J.; Raza S.; Chen X.; Jia C. L. Ewald Summation for Ferroelectric Perovksites with Charges and Dipoles. Comput. Mater. Sci. 2019, 162, 314–321. 10.1016/j.commatsci.2019.03.006.

[ref49] Toukmaji A.; Sagui C.; Board J.; Darden T. Efficient Particle-Mesh Ewald Based Approach to Fixed and Induced Dipolar Interactions. J. Chem. Phys. 2000, 113, 10913–10927. 10.1063/1.1324708.

[ref50] Stamm B.; Lagardére L.; Polack É.; Maday Y.; Piquemal J.-P. A Coherent Derivation of the Ewald Summation for Arbitrary Orders of Multipoles: The Self-Terms. J. Chem. Phys. 2018, 149, 124103 10.1063/1.5044541. 30278683

[ref51] Streeter I.; de Leeuw N. H. Atomistic Modeling of Collagen Proteins in Their Fibrillar Environment. J. Phys. Chem. B 2010, 114, 13263–13270. 10.1021/jp1059984. 20873729 PMC3505825

[ref52] Streeter I.; de Leeuw N. H. A Molecular Dynamics Study of the Interprotein Interactions in Collagen Fibrils. Soft Matter 2011, 7, 3373–3382. 10.1039/c0sm01192d. 23526918 PMC3605786

[ref53] Rieppo J.; Hallikainen J.; Jurvelin J. S.; Kiviranta I.; Helminen H. J.; Hyttinen M. M. Practical Considerations in the Use of Polarized Light Microscopy in the Analysis of the Collagen Network in Articular Cartilage. Microsc. Res. Technol. 2008, 71, 279–287. 10.1002/jemt.20551. 18072283

