# Supplementary material for: Dipolar Relaxation of Water Protons in the Vicinity of a Collagen-like Peptide
Source: J Phys Chem B. 2022 Mar 26;126(13):2538–51. doi: 10.1021/acs.jpcb.2c00052 (PMC8996236; doi:10.1021/acs.jpcb.2c00052)
Supplement: Supplementary file 1 — jp2c00052_si_001.pdf [file jp2c00052_si_001.pdf]

Supporting Information to Dipolar Relaxation of Water Protons in the Vicinity of a Collagen-Like Peptide

Jouni Karjalainen,[*,†] Henning Henschel,[†,‖] Mikko J. Nissi,[‡,†] Miika T. Nieminen,[†,¶,§] and Matti Hanni[*,†,¶,§]

†*Research Unit of Medical Imaging Physics and Technology, University of Oulu, P.O.Box 5000, Oulu, Finland.*

‡*Department of Applied Physics, University of Eastern Finland, Kuopio, Finland.*

¶*Department of Diagnostic Radiology, Oulu University Hospital, Oulu, Finland.*

§*Medical Research Center, University of Oulu and Oulu University Hospital, Oulu, Finland.*

‖*Current address: Department of Medicinal Chemistry, Uppsala University, Box 574, 751 23 Uppsala, Sweden*

E-mail: jouni.karjalainen@oulu.fi; matti.hanni@oulu.fi

Phone: +358 44 3557227

S1 Time-evolution of the expectation values of operators

The expectation value of a quantum-mechanical operator Q can be written as

$$\langle Q \rangle = \mathrm{tr}\{Q\sigma\}, \tag{S1}$$

where σ is the density operator of the system. If Q does not explicitly depend on time we get

$$\frac{\mathrm{d}\langle Q \rangle}{\mathrm{d}t} = \mathrm{tr}\left\{Q\frac{\mathrm{d}\sigma}{\mathrm{d}t}\right\} \tag{S2}$$

The time-evolution of the density operator obeys the Liouville-von Neumann (LvN) equation

$$\frac{\mathrm{d}\sigma}{\mathrm{d}t} = -i[H, \sigma] \tag{S3}$$

We assume that the spin hamiltonian H of the system consists of Zeeman (H_Z) and dipolar [$H_\mathrm{DD}(t)$] interactions, where the Zeeman interaction is time-independent and much stronger than the dipolar interaction. We can split the interaction into two components H_0 and $H_1(t)$ such that the time-average $\langle H_1(t)\rangle_t = 0$:

$$H = H_0 + H_1(t) \tag{S4}$$

$$H_0 = H_\mathrm{Z} + \langle H_\mathrm{DD}(t)\rangle_t \tag{S5}$$

$$\begin{aligned} H_1(t) &= H_\mathrm{DD}(t) - \langle H_\mathrm{DD}(t)\rangle_t \\ &= C\sum_m \left[F_m(t)^* - \langle F_m(t)^*\rangle_t\right] V_m = C\sum_m f_m(t)^* V_m \end{aligned} \tag{S6}$$

In the last step, the dipole-dipole coupling has been decomposed into spatial functions F_m

and spin-operators V_m so that

$$\begin{aligned} H_{\mathrm{DD}}(t) =& C \sum_{m=-2}^{2} F_m(t)^* V_m, \\ C =& -\frac{3\mu_0}{2\pi}\gamma^2\hbar\sqrt{\frac{2\pi}{15}}; \\ F_m(t) =& r_t^{-3} Y_2^m(\theta_t, \phi_t) \end{aligned} \tag{S7}$$

where $r_t = r(t)$, is the length of the internuclear vector and $\theta_t = \theta(t)$ and $\phi_t = \phi(t)$ describe the orientation of the vector with respect to the external magnetic field. The spin operators V_m with $|m| = 0, 1,$ and 2 correspond to zero-, single- and double-quantum coherences, respectively:

$$\begin{aligned} V_0 =& -\frac{I_-S_+ + I_+S_- - 4I_zS_z}{2\sqrt{6}}, \\ V_{\pm 1} =& \pm\frac{1}{2}(I_\pm S_z + I_z S_\pm), \\ V_{\pm 2} =& \frac{1}{2} I_\pm S_\pm. \end{aligned} \tag{S8}$$

The spatial functions F_m contain the normalized 2nd rank spherical harmonics $Y_2^m(\theta, \phi)$:

$$\begin{aligned} Y_2^0(\theta,\phi) =& \frac{1}{4}\sqrt{\frac{5}{\pi}}(3\cos^2\theta - 1), \\ Y_2^{\pm 1}(\theta,\phi) =& \mp\frac{1}{2}\sqrt{\frac{15}{2\pi}}\sin\theta\cos\theta e^{\pm i\phi}, \\ Y_2^{\pm 2}(\theta,\phi) =& \frac{1}{4}\sqrt{\frac{15}{2\pi}}\sin^2\theta e^{\pm i2\phi}. \end{aligned} \tag{S9}$$

Now we proceed to eliminate the time-independent Hamiltonian H_0 from Eq. (S3) with successive unitary transformations. This equals to formally solving the time-dependence of σ due to H_0. In a coordinate system rotating counter-clockwise around the z-axis at angular

velocity ω the LvN Eq. (S3) becomes

$$\frac{\mathrm{d}\tilde{\sigma}}{\mathrm{d}t} = \frac{\mathrm{d}}{\mathrm{d}t}\left\{\exp[i\omega(I_z+S_z)t]\sigma\exp[-i\omega(I_z+S_z)t]\right\}$$
$$= i\left[\omega(I_z+S_z),\tilde{\sigma}\right] - i\left[\tilde{H},\tilde{\sigma}\right] \tag{S10}$$

Next, the Hamiltonian H is transformed to the rotating frame. The Zeeman part is unchanged:

$$\tilde{H}_{\mathrm{Z}} = \exp[i\omega(I_z+S_z)t](\omega_{0I}I_z+\omega_{0S}S_z)\exp[-i\omega(I_z+S_z)t] = (\omega_{0I}I_z+\omega_{0S}S_z). \tag{S11}$$

The hamiltonian of the dipole-dipole couplings in the rotating coordinate frame is simply

$$\tilde{H}_{\mathrm{DD}}(t) = C\sum_m F_m(r,\theta,\phi)^* V_m \exp(im\omega t) \tag{S12}$$

We assume that terms with $m \neq 0$ oscillate fast and can be discarded from the time-averages of the dipole-dipole couplings, so in the rotating frame we have the residual dipolar coupling

$$\langle \tilde{H}_{\mathrm{DD}}(t)\rangle_t = \tilde{H}_{\mathrm{RDC}} \approx C\langle F_0(t)\rangle_t V_0 \tag{S13}$$

By substituting Eqs. (S11)-(S13) into Eq. (S10) we get

$$\frac{\mathrm{d}\tilde{\sigma}}{\mathrm{d}t} = i\left[\omega(I_z+S_z),\tilde{\sigma}\right] - i\left[(\omega_{0I}I_z+\omega_{0S}S_z)+\tilde{H}_{\mathrm{RDC}}+\tilde{H}_1(t),\tilde{\sigma}\right]$$
$$= -i\left[(\omega_{0I}-\omega)I_z+(\omega_{0S}-\omega)S_z+\tilde{H}_{\mathrm{RDC}}+\tilde{H}_1(t),\tilde{\sigma}\right] \tag{S14}$$

If we assume that $\omega = \omega_0$ is the average of the Larmor frequencies ω_{0S} and ω_{0I} and the deviations $\delta_K = (\omega_{0K} - \omega_0)$, $K = I, S$ are small, $|\delta_K| << |\tilde{H}_{RDC}|$ we can neglect the remaining part of the Zeeman interaction.

Similarly to the transformation to the rotating coordinate frame we make a transforma-

tion to remove $H_{\rm RDC}$ from the LvN eq:

$$\begin{aligned}\frac{{\rm d}\tilde{\sigma}^\circ}{{\rm d}t} =&\frac{\rm d}{{\rm d}t}\left\{\exp\left(i\tilde{H}_{\rm RDC}t\right)\tilde{\sigma}\exp\left(-i\tilde{H}_{\rm RDC}t\right)\right\}\\ =&i[\tilde{H}_{\rm RDC},\tilde{\sigma}^\circ]-i[\tilde{H}_{\rm RDC}+\tilde{H}_1^\circ(t),\tilde{\sigma}^\circ]=-i[\tilde{H}_1^\circ(t),\tilde{\sigma}^\circ]\end{aligned}\tag{S15}$$

If we formally integrate the equation above from zero to t, we get

$$\tilde{\sigma}^\circ(t)=\tilde{\sigma}^\circ(0)-i\int_0^t[\tilde{H}_1^\circ(t'),\tilde{\sigma}^\circ(t')]{\rm d}t'.\tag{S16}$$

Substituting this back into Eq. (S15) we have

$$\frac{{\rm d}\tilde{\sigma}^\circ(t)}{{\rm d}t}=-i[\tilde{H}_1^\circ(t),\tilde{\sigma}^\circ(0)]-\int_0^t[\tilde{H}_1^\circ(t),[\tilde{H}_1^\circ(t'),\tilde{\sigma}^\circ(t')]]{\rm d}t'.\tag{S17}$$

Next we take an ensemble average (see Ref. 1) on both sides of the equation above and denote it with an overline:

$$\frac{{\rm d}\overline{\tilde{\sigma}^\circ(t)}}{{\rm d}t}=-\overline{i[\tilde{H}_1^\circ(t),\tilde{\sigma}^\circ(0)]}-\int_0^t\overline{[\tilde{H}_1^\circ(t),[\tilde{H}_1^\circ(t'),\tilde{\sigma}^\circ(t')-\tilde{\sigma}^\circ_{\rm eq}]]}{\rm d}t'\tag{S18}$$

Here we have substituted $\tilde{\sigma}^\circ(t')\to\tilde{\sigma}^\circ(t')-\tilde{\sigma}^\circ_{\rm eq}$ to phenomenologically take into account the finite lattice temperature. We defined $H_1(t)$ so that it has a vanishing time-average. The different members of the ensemble have different random behaviour in $H_1(t)$ but the density operator $\tilde{\sigma}^\circ(0)$ is the same for all the members. For these reasons the first term on the right side in the equation above vanishes. Next step is to inject $H_1(t)$ [Eq. (S6)] into the integrand, with the spin operators transformed as

$$\begin{aligned}\tilde{V}_m^\circ(t)=&\exp\left(i\tilde{H}_{\rm RDC}t\right)V_m\exp\left(-i\tilde{H}_{\rm RDC}t\right)\exp(im\omega t)=V_m^\circ(t)\exp(im\omega t)\\ \tilde{V}_m^{\dagger\circ}(t)=&(-1)^mV_{-m}^\circ(t)\exp(-im\omega t)=V_m^{\dagger\circ}(t)\exp(-im\omega t)\end{aligned}\tag{S19}$$

With these we have

$$\frac{\mathrm{d}\overline{\tilde{\sigma}^{\circ}(t)}}{\mathrm{d}t} = -C^2 \sum_{m,n} \int_0^t \overline{f_m(t)^* f_n(t') \left[\tilde{V}_m^{\circ}(t), \left[\tilde{V}_n^{\dagger\circ}(t'), \tilde{\sigma}^{\circ}(t') - \tilde{\sigma}_{\mathrm{eq}}^{\circ}\right]\right]} \mathrm{d}t' \tag{S20}$$

Now we assume that time-evolution of the density matrix and any spin operators involved is slow as compared to the random functions. With this we can calculate the ensemble averages of the correlation functions $f_m(t)^* f_n(t')$ and the double commutator separately. Using the same assumption we also substitute $\tilde{\sigma}^{\circ}(t') \to \tilde{\sigma}^{\circ}(t)$.

$$\frac{\mathrm{d}\tilde{\sigma}^{\circ}(t)}{\mathrm{d}t} = -C^2 \sum_{m,n} \int_0^t \overline{f_m(t)^* f_n(t')} \left[\tilde{V}_m^{\circ}(t), \left[\tilde{V}_n^{\dagger\circ}(t'), \tilde{\sigma}^{\circ}(t) - \tilde{\sigma}_{\mathrm{eq}}^{\circ}\right]\right] \mathrm{d}t' \tag{S21}$$

Next we go back to the Schrödinger picture with the inverse transformations. First to the rotating frame. The inverse transformations equal to the substitutions

$$\begin{aligned} \tilde{V}_m^{\circ}(t) \to& V_m \exp(im\omega t) \\ \tilde{V}_n^{\dagger\circ}(t') \to& V_n^{\dagger\circ}(t'-t) \exp(-in\omega t') \\ \tilde{\sigma}^{\circ}(t) - \tilde{\sigma}_{\mathrm{eq}}^{\circ} \to& \tilde{\sigma}(t) - \tilde{\sigma}_{\mathrm{eq}} \end{aligned} \tag{S22}$$

and arrive at

$$\begin{aligned} \frac{\mathrm{d}\tilde{\sigma}(t)}{\mathrm{d}t} =& -i[\tilde{H}_{\mathrm{RDC}}, \tilde{\sigma}(t)] - C^2 \sum_{m,n} \int_0^t \overline{f_m(t)^* f_n(t')} \times \\ & \exp(im\omega_0 t) \exp(-in\omega_0 t') \left[V_m, \left[V_n^{\dagger\circ}(t'-t), \tilde{\sigma}(t) - \tilde{\sigma}_{\mathrm{eq}}\right]\right] \mathrm{d}t'. \end{aligned} \tag{S23}$$

We can also go straight to the Schrödinger picture

$$\begin{aligned} \frac{\mathrm{d}\sigma(t)}{\mathrm{d}t} =& -i\left[\omega_0(I_z + S_z) + H_{\mathrm{RDC}}, \sigma(t)\right] + \\ & \exp[-i\omega_0(I_z+S_z)t] \exp\left(-i\tilde{H}_{\mathrm{RDC}}t\right) \frac{\mathrm{d}\tilde{\sigma}^{\circ}(t)}{\mathrm{d}t} \exp\left(i\tilde{H}_{\mathrm{RDC}}t\right) \exp[i\omega_0(I_z+S_z)t] \end{aligned} \tag{S24}$$

Now the operators transform as

$$
\begin{aligned}
\tilde{V}_m^\circ(t) \rightarrow & V_m \\
\tilde{V}_n^{\dagger\circ}(t') \rightarrow & \tilde{V}_n^{\dagger\circ}(t'-t) = V_n^\circ(t'-t)\exp[-in\omega_0(t'-t)] \\
\tilde{\sigma}^\circ(t) - \tilde{\sigma}_{\mathrm{eq}}^\circ \rightarrow & \sigma(t) - \sigma_{\mathrm{eq}}
\end{aligned}
\tag{S25}
$$

and we arrive at

$$
\begin{aligned}
\frac{\mathrm{d}\sigma(t)}{\mathrm{d}t} = & - i\left[\omega_0(I_z+S_z) + H_{\mathrm{RDC}}, \sigma(t)\right] \\
& - C^2 \sum_{m,n} \int_0^t \overline{f_m(t)^* f_n(t')} \exp[-in\omega_0(t'-t)] \times \\
& \left[V_m, \left[V_n^{\dagger\circ}(t'-t), \sigma(t) - \sigma_{\mathrm{eq}}\right]\right] \mathrm{d}t'
\end{aligned}
\tag{S26}
$$

We now assume that the correlation function inside the integral only depends on the absolute value of $t - t' = \tau$, $\overline{f_m(t)^* f_n(t')} = G_{m,n}(|\tau|)$. We also assume that there is some τ_c, such that when $\tau >> \tau_c$, the correlation function vanishes. With this we can extend the integral to infinity.

$$
\begin{aligned}
\frac{\mathrm{d}\sigma(t)}{\mathrm{d}t} = & - i\left[\omega_0(I_z+S_z) + H_{\mathrm{RDC}}, \sigma(t)\right] \\
& - C^2 \sum_{m,n} \int_0^\infty G_{m,n}(|\tau|) \exp(in\omega_0\tau) \left[V_m, \left[V_n^{\dagger\circ}(-\tau), \sigma(t) - \sigma_{\mathrm{eq}}\right]\right] \mathrm{d}\tau
\end{aligned}
\tag{S27}
$$

We would like to represent the second term as a sum of products of Fourier transforms of correlation functions and spin-operators, but the term $V_n^{\dagger\circ}(-\tau) = \exp\left(-i\tilde{H}_{\mathrm{RDC}}\tau\right) V_n^\dagger \exp\left(i\tilde{H}_{\mathrm{RDC}}\tau\right)$ prohibits it still. Even if we defer the exact form of $V_n^{\dagger\circ}(-\tau)$ to be defined later, we can formally finish what we are aiming for: to define relaxation equations for $\langle I_z + S_z \rangle$ and $\langle I_x + S_x \rangle$. For completeness, we'll present the time-evolution of the rotating frame density operator expressed similarly as Eq. (S27). With the assumptions and notation used above

for the Schrödinger picture we get from Eq. (S23)

$$\begin{aligned}\frac{\mathrm{d}\tilde{\sigma}(t)}{\mathrm{d}t} =& -i[\tilde{H}_{\mathrm{RDC}}, \tilde{\sigma}(t)] - C^2 \sum_{m,n} \int_0^\infty G_{m,n}(|\tau|)\times \\ & \exp[i(m-n)\omega_0 t] \exp(in\omega_0\tau) \left[V_m, \left[V_n^{\dagger\circ}(-\tau), \tilde{\sigma}(t) - \tilde{\sigma}_{\mathrm{eq}}\right]\right] \mathrm{d}\tau \end{aligned} \tag{S28}$$

Substituting the time-evolution of the density operator from Eq. (S27) to Eq. (S2) we get

$$\begin{aligned}\frac{\mathrm{d}\langle Q\rangle}{\mathrm{d}t} =& -i\,\mathrm{tr}\left\{Q\left[\omega_0(I_z+S_z) + H_{\mathrm{RDC}}, \sigma(t)\right]\right\} \\ & - C^2 \sum_{m,n} \int_0^\infty G_{m,n}(|\tau|) \exp(in\omega_0\tau)\times \\ & \mathrm{tr}\left\{Q\left[V_m, \left[V_n^{\dagger\circ}(-\tau), \sigma(t) - \sigma_{\mathrm{eq}}\right]\right]\right\} \mathrm{d}\tau \\ =& -i\left\langle\left[Q, \omega_0(I_z+S_z) + H_{\mathrm{RDC}}\right]\right\rangle \\ & - C^2 \sum_{m,n} \int_0^\infty G_{m,n}(|\tau|) \exp(in\omega_0\tau)\times \\ & \left\{\left\langle\left[\left[Q, V_m\right], V_n^{\dagger\circ}(-\tau)\right]\right\rangle - \left\langle\left[\left[Q, V_m\right], V_n^{\dagger\circ}(-\tau)\right]\right\rangle_{\mathrm{eq}}\right\} \mathrm{d}\tau \end{aligned} \tag{S29}$$

In the rotating frame we have

$$\frac{\mathrm{d}\langle Q\rangle_r}{\mathrm{d}t} = \mathrm{tr}\left\{Q\frac{\mathrm{d}\tilde{\sigma}(t)}{\mathrm{d}t}\right\}, \tag{S30}$$

where the subscript r now marks the expectation value in the rotating frame. If we substitute Eq. (S28) and do the same tricks as in the Schrödinger picture above we get

$$\begin{aligned}\frac{\mathrm{d}\langle Q\rangle_r}{\mathrm{d}t} =& -i\left\langle[Q, \tilde{H}_{\mathrm{RDC}}]\right\rangle_r \\ & - C^2 \sum_{m,n} \int_0^\infty G_{m,n}(|\tau|) \exp(in\omega_0\tau) \exp[i(m-n)\omega_0 t]\times \\ & \left\{\left\langle\left[\left[Q, V_m\right], V_n^{\dagger\circ}(-\tau)\right]\right\rangle_r - \left\langle\left[\left[Q, V_m\right], V_n^{\dagger\circ}(-\tau)\right]\right\rangle_{r,\mathrm{eq}}\right\} \mathrm{d}\tau \end{aligned} \tag{S31}$$

From now on we simply assume that we are dealing with a single homonuclear spin-$\frac{1}{2}$ pair I, S in a cylindrically symmetric system. Now we only need to derive the terms which have $m = n$, as will be explained in Sec. S5.2. With these assumptions in place we get for an operator Q:

$$\begin{aligned}\frac{\mathrm{d}\langle Q\rangle}{\mathrm{d}t} =& - i\,\langle[Q, \omega_0(I_z + S_z) + H_{\mathrm{RDC}}]\rangle \\ &- C^2 \sum_m \int_0^\infty G_{m,m}(|\tau|)\exp(im\omega_0\tau)\times \\ &\Big\{\big\langle\big[[Q, V_m], V_m^{\dagger\circ}(-\tau)\big]\big\rangle - \big\langle\big[[Q, V_m], V_m^{\dagger\circ}(-\tau)\big]\big\rangle_{\mathrm{eq}}\Big\}\,\mathrm{d}\tau\end{aligned} \tag{S32}$$

and in the rotating frame

$$\begin{aligned}\frac{\mathrm{d}\langle Q\rangle_r}{\mathrm{d}t} =& - i\Big\langle[Q, \tilde{H}_{\mathrm{RDC}}]\Big\rangle_r - C^2 \sum_m \int_0^\infty G_{m,m}(|\tau|)\exp(im\omega_0\tau)\times \\ &\Big\{\big\langle\big[[Q, V_m], V_m^{\dagger\circ}(-\tau)\big]\big\rangle_r - \big\langle\big[[Q, V_m], V_m^{\dagger\circ}(-\tau)\big]\big\rangle_{r,\mathrm{eq}}\Big\}\,\mathrm{d}\tau\end{aligned} \tag{S33}$$

Here we have also used the property $G_{-m,-m}(|\tau|) = G_{m,m}(|\tau|)$, which will be justified later [Eq. (S67)].

S2 Longitudinal relaxation

The relaxation of the longitudinal magnetization is given by Eq. (S32) when we substitute $Q \to I_z + S_z$:

$$\begin{aligned}\frac{\mathrm{d}\langle I_z + S_z\rangle}{\mathrm{d}t} =& - i\,\langle[I_z + S_z, \omega_0(I_z + S_z) + H_{\mathrm{RDC}}]\rangle \\ &- C^2 \sum_m \int_0^\infty G_{m,m}(|\tau|)\exp(im\omega_0\tau)\times \\ &\Big\{\big\langle\big[[I_z + S_z, V_m], V_m^{\dagger\circ}(-\tau)\big]\big\rangle - \big\langle\big[[I_z + S_z, V_m], V_m^{\dagger\circ}(-\tau)\big]\big\rangle_{\mathrm{eq}}\Big\}\,\mathrm{d}\tau\end{aligned} \tag{S34}$$

Using the property $[I_z + S_z, V_m] = mV_m$ we can simplify Eq. (S34) to

$$\frac{\mathrm{d}\langle I_z + S_z\rangle}{\mathrm{d}t} = -C^2 \sum_m \int_0^\infty G_{m,m}(|\tau|)\exp(im\omega_0\tau)\times \\ \left\{\left\langle \left[mV_m, V_m^{\dagger\circ}(-\tau)\right] \right\rangle - \left\langle \left[mV_m, V_m^{\dagger\circ}(-\tau)\right] \right\rangle_{\mathrm{eq}}\right\}\mathrm{d}\tau \tag{S35}$$

For a spin-1/2 pair we need to calculate the double commutators

$$\left\langle \left[[I_z + I_z, V_m], V_m^{\dagger\circ}(-\tau)\right]\right\rangle = \left\langle \left[mV_m, V_m^{\dagger\circ}(-\tau)\right]\right\rangle. \tag{S36}$$

These, along with the rest of the commutators appearing in the following, were computed with the help of the SpinDynamica[2] package in Mathematica.[3] When $m = 0$, the commutator above is zero. When $m = \pm 1$,

$$\left\langle \left[\pm V_{\pm 1}, V_{\pm 1}^{\dagger\circ}(-\tau)\right]\right\rangle = \frac{1}{8}\cos\left[\frac{1}{2}\sqrt{\frac{3}{2}}C\langle F_0(t)\rangle_t\tau\right]\langle I_z + S_z\rangle \\ \pm i\frac{1}{8}\sin\left[\frac{1}{2}\sqrt{\frac{3}{2}}C\langle F_0(t)\rangle_t\tau\right]\langle I_+S_- + I_-S_+ - 4I_zS_z\rangle \tag{S37}$$

For $m = \pm 2$ we have

$$\left\langle \left[[I_z + S_z, V_{\pm 2}], V_{\pm 2}^{\dagger\circ}(-\tau)\right]\right\rangle = \frac{1}{2}\langle I_z + S_z\rangle \tag{S38}$$

And when we again use the property (S67), we have

$$
\begin{aligned}
\frac{\mathrm{d}\langle I_z + S_z\rangle}{\mathrm{d}t} =& - C^2 \int_0^\infty \mathrm{d}\tau \Bigg\{ \frac{1}{4} G_{1,1}(|\tau|) \cos\left(\frac{\Delta}{2}\tau\right) \cos(\omega_0 \tau) + \\
& \qquad G_{2,2}(|\tau|) \cos(2\omega_0 \tau) \Bigg\} \times \\
& \{\langle I_z + S_z\rangle - \langle I_z + S_z\rangle_{\mathrm{eq}}\} \\
& - C^2 \int_0^\infty \mathrm{d}\tau \frac{1}{4} G_{1,1}(|\tau|) \sin\left(\frac{\Delta}{2}\tau\right) \sin(\omega_0\tau) \times \\
& \{\langle I_+ S_- + I_- S_+ - 4 I_z S_z\rangle - \langle I_+ S_- + I_- S_+ - 4 I_z S_z\rangle_{\mathrm{eq}}\}, \qquad \text{(S39)}
\end{aligned}
$$

where we have used $\sqrt{\frac{3}{2}} C \langle F_0(t)\rangle_t = \Delta$. We'll assume that the correlation functions $G_{m,m}(|\tau|)$ decay fast compared to the oscillation at angular frequency $\Delta/2$, $\tau_c << 1/\Delta$. Therefore we can approximate $\sin\left(\frac{\Delta}{2}\tau\right) \approx 0$ and $\cos\left(\frac{\Delta}{2}\tau\right) \approx 1$ above. With these we have

$$
\frac{\mathrm{d}\langle I_z + S_z\rangle}{\mathrm{d}t} = - \left[\frac{1}{4} J_{1,1}(\omega_0) + J_{2,2}(2\omega_0)\right] \{\langle I_z + S_z\rangle - \langle I_z + S_z\rangle_{\mathrm{eq}}\}, \qquad \text{(S40)}
$$

$$
J_{m,m}(\omega) = C^2 \int_0^\infty G_{m,m}(|\tau|) \cos(\omega\tau) \mathrm{d}\tau \qquad \text{(S41)}
$$

Functions $J_{m,m}(\omega)$ are the spectral densities.

S3 Time-evolution of transverse magnetization without relaxation

The time-evolution of the transverse magnetization for two identical spin-$\frac{1}{2}$ nuclei is given by Eq. S32 with the substitution $Q \to I_x + S_x$:

$$\begin{aligned}\frac{\mathrm{d}\langle I_x + S_x\rangle}{\mathrm{d}t} =& - i \left\langle [I_x + S_x, \omega_0(I_z + S_z) + H_{\mathrm{RDC}}]\right\rangle \\ & - C^2 \sum_m \int_0^\infty G_{m,m}(|\tau|) \exp(im\omega_0\tau) \times \\ & \left\{ \left\langle \left[[I_x + S_x, V_m], V_m^{\dagger\circ}(-\tau) \right] \right\rangle - \left\langle \left[[I_x + S_x, V_m], V_m^{\dagger\circ}(-\tau) \right] \right\rangle_{\mathrm{eq}} \right\} \mathrm{d}\tau \end{aligned} \tag{S42}$$

Including only the first term on the right side of we get

$$\frac{\mathrm{d}\langle I_x + S_x\rangle}{\mathrm{d}t} = - i \left\langle \left[I_x + S_x, \omega_0(I_z + S_z) + \langle C \sum_{m=-2}^{2} (-1)^m F_{-m}(t) V_m \rangle_t \right] \right\rangle \tag{S43}$$

Without justification (at this point) we include only the $m = 0$ term in the RDC. With this we have

$$\frac{\mathrm{d}\langle I_x + S_x\rangle}{\mathrm{d}t} = - \omega_0 \langle I_y + S_y\rangle - \Delta \langle I_y S_z + I_z S_y\rangle, \tag{S44}$$

where we have again used $\sqrt{\frac{3}{2}} C \langle F_0(t)\rangle_t = \Delta$. Now the time-evolution of $\langle I_x + S_x\rangle$ depends on two other operators. We proceed by deriving their time-evolution and the time evolution

of any other operators that pop up during the process:

$$\frac{\mathrm{d}\langle I_y + S_y\rangle}{\mathrm{d}t} = \omega_0(I_x + S_x) + \Delta(I_xS_z + I_zS_x) \tag{S45}$$

$$\frac{\mathrm{d}\langle I_xS_z + I_zS_x\rangle}{\mathrm{d}t} = -\,\omega_0\langle I_yS_z + I_zS_y\rangle - \frac{\Delta}{4}\langle I_y + S_y\rangle \tag{S46}$$

$$\frac{\mathrm{d}\langle I_yS_z + I_zS_y\rangle}{\mathrm{d}t} = \omega_0\langle I_xS_z + I_zS_x\rangle + \frac{\Delta}{4}\langle I_x + S_x\rangle \tag{S47}$$

Eqs. S44-S47 define a closed system of first-order homogeneous differential equations. If we define a column vector

$$v = \left(\langle I_x + S_x\rangle, \langle I_y + S_y\rangle, \langle I_xS_z + I_zS_x\rangle, \langle I_yS_z + I_zS_y\rangle\right)^{\mathrm{T}}, \tag{S48}$$

we can write the problem as a matrix equation

$$\frac{\mathrm{d}v}{\mathrm{d}t} = Av, \quad A = \begin{pmatrix} 0 & -\omega_0 & 0 & -\Delta \\ \omega_0 & 0 & \Delta & 0 \\ 0 & -\frac{\Delta}{4} & 0 & -\omega_0 \\ \frac{\Delta}{4} & 0 & \omega_0 & 0 \end{pmatrix} \tag{S49}$$

Now the general solution of this equation is

$$v'(t) = \sum_{k=1}^{4} c_k v_k \exp(\lambda_k t) \tag{S50}$$

where λ_k and v_k are the eigenvalues and -vectors of A.[4] Solving the eigenvalues and -vectors and finding the specific solutions was done with Wolfram Mathematica 11.2.[3] In this partic-

ular case we have

$$\lambda_1 = -\,i\left(\frac{\Delta}{2} - \omega_0\right), \lambda_2 = i\left(\frac{\Delta}{2} - \omega_0\right), \lambda_3 = -i\left(\frac{\Delta}{2} + \omega_0\right), \lambda_4 = i\left(\frac{\Delta}{2} + \omega_0\right)$$
$$v_1 = (-2i, -2, i, 1)^T, \quad v_2 = (2i, -2, -i, 1)^T$$
$$v_3 = (-2i, 2, -i, 1)^T, \quad v_4 = (2i, 2, i, 1)^T \tag{S51}$$

For specific solutions one needs to determine some initial conditions. If for example at $t = 0$ the magnetization is along the x-axis, then we have $v'(0) = (\langle I_x + S_x\rangle(0), 0, 0, 0)^{\mathrm{T}}$ and $c_1 = c_3 = -c_2 = -c_4 = \frac{i}{8}\langle I_x + S_x\rangle(0)$. The x-component of the magnetization is given by taking the first component of each vector v_k:

$$\begin{aligned}\langle I_x + S_x\rangle =& \sum_k c_k v_{k1} \exp(\lambda_k t)\\ =& \frac{i}{8}\langle I_x + S_x\rangle(0)\left\{(-2i)\exp\left[-i\left(\frac{\Delta}{2} - \omega_0\right)t\right] - (2i)\exp\left[i\left(\frac{\Delta}{2} - \omega_0\right)t\right] + \right.\\ &\left.(-2i)\exp\left[-i\left(\frac{\Delta}{2} + \omega_0\right)t\right]\right\} - (2i)\exp\left[i\left(\frac{\Delta}{2} + \omega_0\right)t\right]\Bigg\}\\ =& \langle I_x + S_x\rangle(0)\cos\left(\frac{\Delta}{2}t\right)\cos(\omega_0 t)\end{aligned} \tag{S52}$$

The result contains the familiar Larmor frequency oscillation but it is modulated by another oscillation resulting from the dipolar coupling.

S4 Relaxation of transverse magnetization in the rotating frame

Now we compute the time-evolution of the expectation value of the operator $I_{x_r} + S_{x_r}$, where x_r is an axis of the frame rotating at the Larmor frequency around z-axis. At $t = 0$ the x-axis of the laboratory frame and the x_r-axis of the rotating frame coincide, hence $\langle I_x + S_x\rangle(0) = \langle I_{x_r} + S_{x_r}\rangle(0)$. If we assume that the correlation functions decay fast enough,

so that $1/\tau_c >> \Delta$, repeated application of Eq. (S33) to $I_{x_r}+S_{x_r}$ and the other spin operators that emerge in the process, produces the matrix equation

$$\frac{\mathrm{d}v}{\mathrm{d}t} = Av, \quad A = \begin{pmatrix} a & c & 0 & -d \\ -c & a & d & 0 \\ 0 & d/4 & b & c \\ -d/4 & 0 & -c & b \end{pmatrix}, \tag{S53}$$

$$\begin{aligned} a =& -\frac{1}{8}\left[3J_{0,0}(0) + 5J_{1,1}(\omega_0) + 2J_{2,2}(2\omega_0)\right], \\ b =& -\frac{1}{8}\left[3J_{0,0}(0) + J_{1,1}(\omega_0) + 2J_{2,2}(2\omega_0)\right], \\ c =& -\frac{1}{8}\left[L_{1,1}(\omega_0) + 2L_{2,2}(2\omega_0)\right], \\ d =& \Delta. \end{aligned} \tag{S54}$$

Here, $J_{m,m}(\omega)$ is again the spectral density as defined in (S41). In the simulation cases considered in the main text, the relative difference between $J(\omega_0 \pm \Delta/2)$ and $J(\omega_0 \pm \Delta/2)$ is commonly on the order of 10^{-6} or even several orders of magnitude smaller and we have approximated that $J(\omega_0 \pm \Delta/2) \approx J(\omega_0)$. The other functions appearing above are

$$L_{mm}(\omega) = L_{mm}(\omega) = C^2 \int_0^{\infty} G'_{mm}(|\tau|)\sin(\omega\tau)\mathrm{d}\tau \tag{S55}$$

The general solution v' to $\frac{\mathrm{d}v}{\mathrm{d}t} = Av$ is of the same form as in Eq. (S50). The special solution in which $v(t=0) = (\langle I_x + S_x\rangle(0), 0, 0, 0)^{\mathrm{T}}$ gives the x_r-component evolution in the case the magnetization is initially along the x_r axis. We end up with three cases:

1) $4\Delta^2 - J_{1,1}(\omega_0)^2 > 0$**:**

$$\begin{aligned}\langle I_{x_r} + S_{x_r}\rangle =& \langle I_x + S_x\rangle(0) \exp\left\{-\frac{1}{8}t\left[3J_{0,0}(0) + 3J_{1,1}(\omega_0) + 2J_{2,2}(2\omega_0)\right]\right\} \times \\ & \cos\left\{-\frac{1}{8}\left[L_{1,1}(\omega_0) + 2L_{2,2}(2\omega_0)\right]t\right\} \times \\ & \left\{\cos\left(\frac{1}{4}t\sqrt{4\Delta^2 - J_{1,1}(\omega_0)^2}\right) - \frac{J_{1,1}(\omega_0)\sin\left(\frac{1}{4}t\sqrt{4\Delta^2 - J_{1,1}(\omega_0)^2}\right)}{\sqrt{4\Delta^2 - J_{1,1}(\omega_0)^2}}\right\}\end{aligned} \tag{S56}$$

2) $4\Delta^2 - J_{1,1}(\omega_0)^2 < 0$**:**

$$\begin{aligned}\langle I_{x_r} + S_{x_r}\rangle =& \langle I_x + S_x\rangle(0) \exp\left\{-\frac{1}{8}t\left[3J_{0,0}(0) + 3J_{1,1}(\omega_0) + 2J_{2,2}(2\omega_0)\right]\right\} \times \\ & \cos\left\{-\frac{1}{8}\left[L_{1,1}(\omega_0) + 2L_{2,2}(2\omega_0)\right]t\right\} \times \\ & \left\{\cosh\left(\frac{1}{4}t\sqrt{J_{1,1}(\omega_0)^2 - 4\Delta^2}\right) - \frac{J_{1,1}(\omega_0)\sinh\left(\frac{1}{4}t\sqrt{J_{1,1}(\omega_0)^2 - 4\Delta^2}\right)}{\sqrt{J_{1,1}(\omega_0)^2 - 4\Delta^2}}\right\}\end{aligned} \tag{S57}$$

3) $4\Delta^2 - J_{1,1}(\omega_0)^2 = 0$**:**

$$\begin{aligned}\langle I_{x_r} + S_{x_r}\rangle =& \langle I_x + S_x\rangle(0) \exp\left\{-\frac{1}{8}t\left[3J_{0,0}(0) + 3J_{1,1}(\omega_0) + 2J_{2,2}(2\omega_0)\right]\right\} \times \\ & \cos\left\{-\frac{1}{8}\left[L_{1,1}(\omega_0) + 2L_{2,2}(2\omega_0)\right]t\right\}\left[1 - t\frac{J_{1,1}(\omega_0)}{4}\right]\end{aligned} \tag{S58}$$

We see that all the solutions 1-3 are oscillatory, with angular frequency $-\frac{1}{8}\left[L_{1,1}(\omega_0) + 2L_{2,2}(2\omega_0)\right]$. Apart from that, case 1 represents a damped oscillation. In case 2 there is no oscillation and it represents a biexponential decay. The less practical case 3 has a distinct form with a combined linear and exponential decay. In the case RDC is negligible, $\Delta << J_{1,1}(\omega_0)$, we

have the form

$$\begin{aligned}\langle I_{x_r} + S_{x_r}\rangle =& \langle I_x + S_x\rangle(0) \exp\left\{-\frac{1}{8} t \left[3J(0) + 5J(\omega_0) + 2J(2\omega_0)\right]\right\} \\ &\times \cos\left\{-\frac{1}{8}\left[L_{1,1}(\omega_0) + 2L_{2,2}(2\omega_0)\right] t\right\}\end{aligned} \tag{S59}$$

S5 Orientation-dependency of relaxation rates

S5.1 Rotation of the random functions in dipole-dipole coupling

The spherical harmonics in F_m [Eq. (4) in the main text] transform in rotations around the origin from (θ', ϕ') to (θ, ϕ) as

$$Y_2^m(\theta, \phi) = \sum_n D_{mn}^{(2)}(\alpha, \beta, \gamma)^* Y_2^n(\theta', \phi') \tag{S60}$$

Here, $D_{mn}(\alpha, \beta, \gamma)^*$ is the complex conjugate of a Wigner-D-matrix element. The functions f_m [Eq. (14) in the main text] also transform in rotations like spherical harmonics, since

$$\begin{aligned}f_m(r, \theta, \phi) =& r^{-3} Y_2^m(\theta, \phi) - \langle r^{-3} Y_2^m(\theta, \phi)\rangle_t \\ =& r^{-3} \sum_n D_{mn}^{(2)}(\alpha, \beta, \gamma)^* Y_2^n(\theta', \phi') \\ & - \left\langle r^{-3} \sum_n D_{mn}^{(2)}(\alpha, \beta, \gamma)^* Y_2^n(\theta, \phi)\right\rangle_t \\ =& \sum_n D_{mn}^{(2)}(\alpha, \beta, \gamma)^* \left\{r^{-3} Y_2^n(\theta', \phi') - \langle r^{-3} Y_2^n(\theta', \phi')\rangle_t\right\} \\ =& \sum_n D_{mn}^{(2)}(\alpha, \beta, \gamma)^* f_n(r, \theta', \phi')\end{aligned} \tag{S61}$$

The result follows from r being independent of orientation and the rotation being independent of time.

S5.2 Rotation of correlation functions and spectral densities

For the dipole-dipole couplings of spin pairs KL and MN we can write the correlation function $G_{mn}(|\tau|) = \overline{f_m(r_0,\theta_0,\phi_0)^* f_n(r_\tau,\theta_\tau,\phi_\tau)}$, where subscripts are used for different time points, e.g. $\theta_t = \theta(t)$. Now the correlation functions transform in rotations as

$$\begin{aligned} G_{mn}(|\tau|) &= \overline{f_m(r_0,\theta_0,\phi_0)^* f_n(r_\tau,\theta_\tau,\phi_\tau)} \\ &= \sum_{m',n'} D^{(2)}_{mm'}(\alpha,\beta,\gamma) D^{(2)}_{nn'}(\alpha,\beta,\gamma)^* \overline{f_{m'}(r_0,\theta'_0,\phi'_0)^* f_{n'}(r_\tau,\theta'_\tau,\phi'_\tau)}, \\ &= \sum_{m',n'} D^{(2)}_{mm'}(\alpha,\beta,\gamma) D^{(2)}_{nn'}(\alpha,\beta,\gamma)^* G'_{m'n'}(|\tau|) \end{aligned} \tag{S62}$$

Here we have assumed that the rotation is the same for all spin pairs.

Let's assume now that in the primed coordinate frame (r,θ',ϕ') the relative motion of the pair of nuclei has on average uniaxial symmetry with respect to the z'-axis. Then the result of the rotation to some other coordinate frame can not depend on angles α or γ, since they don't change the orientation of the z'-axis. This means that only the Wigner D-matrix elements which have $m = n$ and $m' = n'$ can be involved in the rotation formula. With this we arrive at

$$G_{mm}(|\tau|) = \sum_{m'=-2}^{2} \left[d^{(2)}_{mm'}(\beta)\right]^2 G'_{m'm'}(|\tau|), \tag{S63}$$

where $d^{(2)}_{mm'}(\beta)$ are the Wigner small d-matrix elements.

From Eq. (S63) one can obtain a formula for rotating the spectral densities by multiplying both sides with $\cos(\omega\tau)$ and integrating over τ:

$$J_{mm}(\omega) = \sum_{m'=-2}^{2} \left[d^{(2)}_{mm'}(\beta)\right]^2 J_{m'm'}(\omega) \tag{S64}$$

Similarly, using $\sin(\omega\tau)$ in place of $\cos(\omega\tau)$ we get

$$L_{mm}(\omega) = \sum_{m'=-2}^{2} \left[d^{(2)}_{mm'}(\beta)\right]^2 L_{m'm'}(\omega) \tag{S65}$$

One additional result will be important. That is

$$\begin{aligned} G_{-m,-m}(|\tau|) =& \overline{f_{-m}(r_0,\theta_0,\phi_0)^* f_{-m}(r_\tau,\theta_\tau,\phi_\tau)} \\ =& \overline{f_m(r_0,\theta_0,\phi_0) f_m(r_\tau,\theta_\tau,\phi_\tau)^*} \end{aligned} \tag{S66}$$

Due to the assumptions that we are looking at steady-state correlation functions and that they depend only on the absolute value $|\tau|$ we get

$$G_{-m,-m}(|\tau|) = G_{m,m}(|\tau|), \tag{S67}$$

from which it follows that

$$J_{-m,-m}(\omega) = J_{m,m}(\omega), \tag{S68}$$

With the rotation formulas for the spectral densities [Eq. (S64) and the RDCs [Eq. (S60)] we can transform the relaxation equations (S40) and (S56)-(S59) from the PAS of the uniaxially symmetric system to any other orientation.

S6 Radial distribution functions between water and the peptide

In Sec. 4.1 of the main text we defined the first hydration layer as the water molecules which have any atom closer than 3.5 Å from any peptide atom. This rather rough definition of the hydration layer is certainly not the only possible choice, but suffices in our purpose, where we aim to simply see how the ^{1}H relaxation rates resulting from ^{1}H-^{1}H dipole-dipole couplings

are affected by the presence of the peptide.

To see how accurate our choice of hydration layer thickness is, we can take a look at the radial distribution functions (RDFs) between water and peptide atoms (Fig. S1). From Fig. S1a we see that there is a small local maximum at $r = 1.8$ Å. This corresponds to the water hydrogen (oxygen) atoms, which form a hydrogen bond with the peptide oxygen (hydrogen) atoms, as can be seen from Fig. S1b-c. The next peaks further away from the peptide in Fig. S1b-c correspond to atom pairs which are not H-bonded. In Fig. S1a their cumulative effect is seen as a rise starting from $r = 2.2$ Å and then a change of slope at $r = 2.7$ Å in the water-peptide RDF, until the next "step" is met at $r = 3.7$ Å. The behaviour in Fig. S1a at $r < 3.7$ Å is seen to result from the local maxima seen in the water oxygen RDFs with peptide N,O and H atoms, as well as from the local maximum of the RDF for water hydrogens and peptide oxygens (Fig. S1c). In summary, our criterion for the hydration layer, $r < 3.5$ Å, contains the first two peaks of the O-H and H-O RDFs as well as the first peaks of the O-N and O-O RDFs. Our definition for the second hydration layer probably contains contributions from the second hydration layer as well as beyond that.

S7 Justification of cylindrical symmetry

The components of the RDC tensor were computed in the PAS of peptide inertia for the intra- and intermolecular couplings of all water molecules. By diagonalizing the tensor and comparing its eigenvectors to the axes of the aforementioned PAS we were able to see if the dipolar couplings have uniaxial symmetry and if the symmetry axes coincide with the PAS of the peptide inertia tensor. For the intramolecular couplings in water and the couplings between the water and the peptide, the eigenvector corresponding to the largest eigenvalue of the RDC and the eigenvalue corresponding to the smallest eigenvalue of the inertia tensor have less than 4 degree angle between them. Also for all couplings combined the angle is less than 5 degrees. This means the correspondence between the two PASs is very good. For

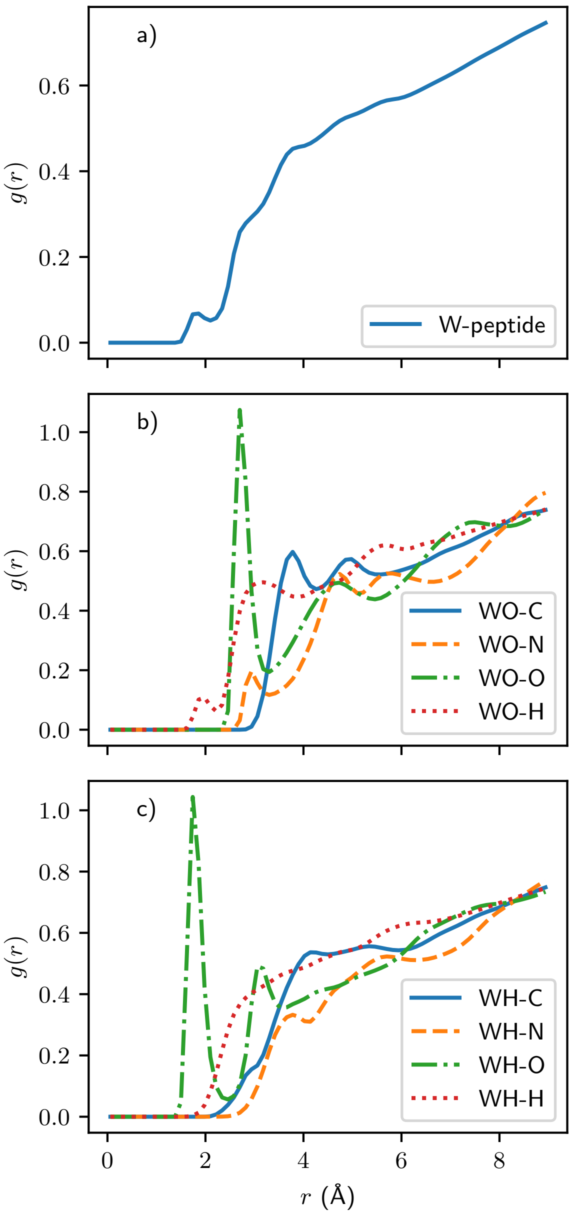

Figure S1: Radial distribution functions (RDFs) $g(r)$ between water and peptide atoms computed from the simulated trajectory. a) W-protein is the RDF between all water atoms and all peptide atoms. b) RDFs between water oxygen atoms (WO) and different peptide atoms (C, N, O, H). c) RDFs between water hydrogen atoms (WH) and different peptide atoms.

the intermolecular couplings between water molecules the angle is larger, approximately 27 degrees, but as discussed in the previous section, the RDC estimate for these couplings is lacking the long-range contributions.

For a diagonal tensor $\mathbf{D}$, the asymmetry $\eta = \frac{D_{11}-D_{22}}{D_{33}}$ gives its deviation from cylindrical symmetry with respect to axis 3. Here, the asymmetry was computed in the PAS of the inertia tensor of the peptide to be able to estimate its error with the similar methods as for the other quantities. Vanishing asymmetry would indicate that the RDC tensor is symmetric with respect to rotations around the main axis of the PAS of the inertia tensor and that the PASs of the inertia and RDC coincide, essentially satisfying the assumption that the RDC tensor is symmetric with respect to rotations around ϕ'. The RDC tensor asymmetry was 0.3 ± 0.2, -0.2 ± 0.05 and 0.00 ± 0.02 for the intramolecular, intermolecular and water-peptide ^{1}H-^{1}H couplings, respectively. For all couplings combined the asymmetry was 0.30 ± 0.07. The asymmetry of the residual intramolecular couplings is fairly high, but also its errors are relatively large. We acknowledge these and consider the cylindrical symmetry as an approximation or a rotational average around the long axis of the peptide.

The assumption of cylindrical symmetry greatly simplifies the theoretical treatment of the dipolar relaxation. The main consequence of the assumption is that correlation functions $G_{mn}(|\tau|)$ with $m \neq n$ are neglected. We have computed the spectral densities $J'_{mn}(0)$ for all m, n and found that the magnitude of the smallest $J'_{mm}(0)$ was still more than eight times the largest $J'_{mn}(0), m \neq n$. Furthermore, the $m \neq n$ components only contribute to cross-relaxation between the different spin operators, which further reduces their effect.

S8 Anisotropy in spectral densities

The amount of anisotropy in each of the spectral densities varies with the type of coupling and the water selection (Table S2). We computed the fraction of the anisotropic part in the

spectral densities and relaxation rates as

$$g(Q) = 1 - Q_{\mathrm{min}}/Q_{\mathrm{max}}, \tag{S69}$$

where Q is replaced with respective spectral density or relaxation rate. The combined spectral densities were anisotropic in the first hydration layer (1st h.l. all), but already in the second hydration layer (2nd h.l. all) the anisotropy falls below the estimated error in fitting the TCFs. The largest anisotropies are found in the spectral densities of the couplings between water and the peptide. In the first hydration layer they range from 14.5 % to 17.9 %. In the second hydration layer $J_{1,1}(\omega_0)$ appears to be isotropic and $J_{0,0}(0)$, as well as $J_{2,2}(2\omega_0)$ have anisotropies of 15 % and 7 %, respectively. When all water molecules are taken into account, the spectral densities of couplings with the peptide also appear to be anisotropic, with anisotropy ranging from 8.2 % in $J_{1,1}(\omega_0)$ to 13.6 % in $J_{2,2}(2\omega_0)$. However, the magnitude of these spectral densities (Table S1) is very small compared to the ones coming from the intramolecular couplings, and the latter dominate the overall orientation dependence of the relaxation rates.

The spectral densities of the intramolecular couplings are anisotropic only in the first hydration layer, where the fraction of the anisotropic part ranges from 9 % in $J_{1,1}(\omega_0)$ to 15 % in $J_{2,2}(2\omega_0)$. Already in the second hydration layer the spectral densities appear to be isotropic or the anisotropy falls within the error from fitting the TCFs. All spectral densities of the intermolecular coupling between water molecules were either clearly isotropic [$J_{1,1}(\omega_0)$ and $J_{2,2}(2\omega_0)$], or weakly anisotropic [$J_{0,0}(0)$] in the first and second hydration layers. The spectral densities of couplings between all water molecules (water inter) were isotropic.

References

(1) Goldman, M. Formal Theory of Spin–Lattice Relaxation. *J. Magn. Reson.* **2001**, *149*, 160–187.

Table S1: Spectral densities computed at $\nu_0 = \frac{\omega_0}{2\pi} = 400$ MHz. For the peptide system, the values were computed in the PAS of the inertia tensor ($\beta = 0$). For the bulk system axes of the coordinate system were aligned along the vertices of the simulation box. The errors represent 95 % confidence intervals computed with the bootstrap method. See Sec. 4.1 in the main text for an explanation of the labels.

	$J'_{0,0}(0)$ (1/s)	$J'_{1,1}(\omega_0)$ (1/s)	$J'_{2,2}(2\omega_0)$ (1/s)
1st h.l. all	0.84 ± 0.02	0.85 ± 0.02	0.91 ± 0.02
1st h.l., intra	0.44 ± 0.02	0.43 ± 0.01	0.52 ± 0.02
1st h.l., inter	0.223 ± 0.002	0.232 ± 0.002	0.233 ± 0.002
1st h.l., pept. H	0.194 ± 0.003	0.197 ± 0.003	0.157 ± 0.002
2nd h.l. all	$0.352^{+0.009}_{-0.008}$	$0.361^{+0.006}_{-0.007}$	0.373 ± 0.005
2nd h.l., intra	$0.165^{+0.007}_{-0.005}$	0.169 ± 0.004	0.175 ± 0.004
2nd h.l., inter	0.172 ± 0.001	$0.1765^{+0.0010}_{-0.0009}$	0.180 ± 0.001
2nd h.l., pept. H	$0.0176^{+0.0009}_{-0.0005}$	$0.0219^{+0.0007}_{-0.0006}$	0.0218 ± 0.0003
water intra	0.1462 ± 0.0009	0.1468 ± 0.0008	$0.1479^{+0.0010}_{-0.0009}$
water inter	0.1044 ± 0.0003	$0.1045^{+0.0003}_{-0.0002}$	$0.1043^{+0.0003}_{-0.0002}$
water, pept. H	$0.00571^{+0.00008}_{-0.00009}$	$0.00658^{+0.00009}_{-0.00008}$	0.00550 ± 0.00005
bulk intra	0.139 ± 0.002	0.140 ± 0.002	0.141 ± 0.002
bulk inter	$0.0928^{+0.0006}_{-0.0007}$	0.0930 ± 0.0007	0.0928 ± 0.0007

(2) Bengs, C.; Levitt, M. H. SpinDynamica: Symbolic and Numerical Magnetic Resonance in a Mathematica Environment. *Magn. Reson. Chem.* **2018**, *56*, 374–414.

(3) Mathematica, Version 11.2. Wolfram Research, Inc., 2019.

(4) Kreyszig, E. *Advanced Engineering Mathematics*, 8th ed.; Wiley: New York, 1999.

Table S2: The fraction of the anisotropic part $g(Q) = 1 - Q_{\mathbf{min}}/Q_{\mathbf{max}}$ and its 95 % confidence interval for the spectral densities at $\nu_0 = \frac{\omega_0}{2\pi} = 400$ MHz. The errors represent 95 % confidence intervals computed with the bootstrap method. See Sec. 4.1 in the main text for an explanation of the labels.

	$g[J_{0,0}(0)]$	$g[J_{1,1}(\omega_0)]$	$g[J_{2,2}(2\omega_0)]$
1st h.l. all	0.06 ± 0.02	0.03 ± 0.01	0.06 ± 0.02
1st h.l., intra	0.13 ± 0.03	0.09 ± 0.02	0.15 ± 0.03
1st h.l., inter	0.031 ± 0.005	0.002 ± 0.003	0.017 ± 0.004
1st h.l., pept. H	$0.145^{+0.009}_{-0.008}$	0.102 ± 0.004	0.179 ± 0.007
2nd h.l. all	0.04 ± 0.02	0.02 ± 0.01	$0.04^{+0.01}_{-0.02}$
2nd h.l., intra	0.04 ± 0.03	0.02 ± 0.02	0.04 ± 0.02
2nd h.l., inter	$0.031^{+0.004}_{-0.003}$	0.009 ± 0.002	0.024 ± 0.003
2nd h.l., pept. H	$0.15^{+0.02}_{-0.03}$	$0.00^{+0.02}_{-0.01}$	$0.07^{+0.02}_{-0.03}$
water intra	$0.008^{+0.006}_{-0.005}$	$0.003^{+0.004}_{-0.003}$	0.008 ± 0.005
water inter	0.0012 ± 0.0010	0.0013 ± 0.0006	0.0019 ± 0.0009
water, pept. H	$0.028^{+0.009}_{-0.01}$	0.082 ± 0.004	0.101 ± 0.007
bulk intra	0.01 ± 0.01	$0.004^{+0.011}_{-0.008}$	$0.01^{+0.02}_{-0.01}$
bulk inter	0.000 ± 0.003	0.001 ± 0.002	0.001 ± 0.003
